# Relative Efficacy of Vitamin D_2_ and Vitamin D_3_ in Improving Vitamin D Status: Systematic Review and Meta-Analysis

**DOI:** 10.3390/nu13103328

**Published:** 2021-09-23

**Authors:** Rakesh Balachandar, Raghu Pullakhandam, Bharati Kulkarni, Harshpal Singh Sachdev

**Affiliations:** 1 ICMR-National Institute of Occupational Health, Ahmedabad 380016, India; balachandar.rakesh@gmail.com; 2 ICMR-National Institute of Nutrition, Hyderabad 500007, India; raghu_nin2000@yahoo.com; 3 Sitaram Bhartia Institute of Science and Research, New Delhi 110016, India; hpssachdev@gmail.com

**Keywords:** ergocalciferol, cholecalciferol, parathyroid hormone, vitamin D, vitamin D_2_, vitamin D_3_

## Abstract

Background: Widespread prevalence of vitamin D deficiency has been documented globally. Commonly used interventions to address this deficiency include supplementation and/or fortification with either ergocalciferol (vitamin D_2_) or cholecalciferol (vitamin D_3_), but the relative efficacy of these two vitamers is unclear. The current study aimed to evaluate the relative efficacy of ergocalciferol (vitamin D_2_) and cholecalciferol (vitamin D_3_) for raising the serum levels of vitamin D metabolites and functional indicators including serum parathyroid (PTH) levels, isometric muscle strength, hand grip strength and bone mineral density. Methods: Randomized and non-randomized controlled studies evaluating relative efficacy of ergocalciferol and cholecalciferol were systematically reviewed to synthesize quantitative and qualitative evidence as per the recommendations of according to “Preferred Reporting Items for Systematic reviews and Meta-analysis” guidelines. Search terms were constructed on the basis of the “participants”, “intervention”, “control”, “outcome” and “study type” (PICOS) strategy to systematically search the popular electronic databases. Relevant data from studies meeting inclusion and exclusion criteria were extracted and analyzed. Meta-regression, subgroup and sensitivity analyses were performed to investigate the influence of study-level characteristics including intervention dosage, frequency of dosing, interval between the last dose and test for outcome assessment, participant characteristics and analytical methods. Results: Apparently healthy human participants (*n* = 1277) from 24 studies were included for meta-analysis. The quantitative analysis suggested higher efficacy of cholecalciferol than ergocalciferol in improving total 25(OH)D (mean difference: 15.69, 95%CI: 9.46 to 21.93 nmol/L) and reducing PTH levels, consistently across variable participant demographics, dosage and vehicle of supplementation. Meta-regression suggested smaller differences in the efficacy of cholecalciferol and ergocalciferol at lower doses. Average daily dose was the single significant predictor of effect size, as revealed by multivariate meta-regression analysis. Conclusions: Compared to ergocalciferol, cholecalciferol intervention was more efficacious in improving vitamin D status (serum levels of total 25(OH)D and 25(OH)D_3_) and regulating PTH levels, irrespective of the participant demographics, dosage and vehicle of supplementation.

## 1. Introduction

Vitamin D is a group of fat-soluble vitamins traditionally recognized for its role in maintaining the homeostasis of calcium and phosphorous. Vitamin D commonly occurs in two forms: vitamin D_2_ and vitamin D_3_. Vitamin D_3_, also known as cholecalciferol, is synthesized de novo in the skin on exposure to ultraviolet-B radiation, and it is also available from animal source foods [1]. Vitamin D_2_ (ergocalciferol) is obtained from plants, particularly mushrooms and yeast. Structurally, vitamin D_2_ differs from vitamin D_3_ in having a double bond between C_22_ and C_23_ and a methyl group at C_24_ [2]. Vitamin D_2_ and vitamin D_3_ undergo two sequential enzymatic hydroxylation reactions to be biologically active. The first hydroxylation occurs in the liver, which results in conversion of vitamin D_2_ and vitaminD_3_ to 25(OH)D_2_ and 25(OH)D_3_, respectively. The second reaction occurs in the kidneys, wherein 25(OH)D_2_ and 25(OH)D_3_ are converted to their respective biologically active forms 1,25 dihydroxy vitamin D_2_ and 1,25 dihydroxy vitamin D_3_ [1]. As the circulating levels of total 1,25(OH)_2_D are homeostatically regulated, serum total 25(OH)D is considered to reflect the vitamin D status [3]. Both ergocalciferol and cholecalciferol are reported to exhibit similar potency in terms of their ability to cure vitamin D deficiency rickets [1].

Vitamin D deficiency is currently a global health problem. It is estimated that about 30% of adults have vitamin D deficiency (serum 25(OH)D < 50 nmol/L) and about 60% have insufficiency (serum 25(OH)D 50–75 nmol/L) [4]. The underlying reasons are probably multi-factorial including socio-cultural practices of avoiding sun exposure, dietary restrictions, environmental pollution, increased prevalence of obesity and genetic causes [5,6]. Tropical countries (such as India) with abundant sunlight are no exception, as high prevalence of vitamin D deficiency (30–80%) has been reported among adults [4] as well as among children and adolescents [7]. In addition to its classic functions, recent research also suggests the potential benefits of vitamin D in diabetes mellitus, metabolic syndrome, malignancy, hypertension, cardiovascular illness and neuropsychiatric disorders [8,9,10]. Alleviating vitamin D deficiency is, therefore, of public health significance.

Therapeutic supplementation and food fortification are the commonly used strategies for improving vitamin D status. Multiple intervention studies have demonstrated the efficacy of vitamin D (vitamin D_2_ or vitamin D_3_) supplementation, either as a single large bolus or given in divided doses by oral and parenteral routes, in raising the serum levels of the respective forms of 25(OH)D to varying levels [11,12]. Though both the vitamers increase the serum or plasma total 25(OH)D levels, their relative efficacy remains unclear. The national guidelines on food fortification in many countries including India do not specify the choice of the vitamin D fortificant and recommend a similar dose of vitamin D_2_ and D_3._ This is based on the assumption that the two vitamers have similar biological activities and are equally potent [13]. However, the equivalent potency of the two forms of vitamin D is based on studies on prevention and cure of rickets with either of the two vitamers in experimental animals and humans [14]. However, in order to enhance the effectiveness of the food fortification program, there is a need to evaluate the relative efficacy of these two vitamers in improving the serum vitamin D levels and influencing parathyroid hormone (PTH), a biomarker of bone mineral metabolism.

Previous systematic review concluded that cholecalciferol is more efficacious than ergocalciferol in raising the serum levels of total 25(OH)D [15]. As the metabolites of vitamin D_2_ and vitamin D_3_ are structurally different, studies comparing the efficacy of these two forms should ideally also estimate the individual metabolites: 25(OH)D_2_ and 25(OH)D_3_. However, some of the studies included in the above meta-analysis did not report this crucial information [16,17,18,19]. Further, the relative effect of these two vitamers on serum PTH levels was also not evaluated. There is thus a need for a systematic review to evaluate the relative efficacy of vitamin D_2_ and D_3_ in raising the serum levels of different metabolites of vitamin D (total 25(OH)D, 25(OH)D_2_ and 25(OH)D_3_) and in modulating calcium homeostasis, as measured by serum PTH levels. Further, it is imperative to examine the relative efficacy of these two vitamers in relation to the baseline vitamin D status for better targeting of the intervention and in relation to the intervention dosage, frequency of dosing and duration of supplementation in order to understand their relative efficacy at different dosage regimes and during short-term and long-term use. Information on these aspects would be helpful for public health policy and practice.

We, therefore, conducted this systematic review to evaluate the relative efficacy of ergocalciferol and cholecalciferol supplementation in raising the serum levels of vitamin D metabolites (total 25(OH)D, 25(OH)D_2_ and 25(OH)D_3_) and functional indicators such as serum PTH, isometric muscle strength, hand grip strength and bone mineral density. Additionally, we explored the influence of various study-level characteristics including the dose of the intervention, dosing frequency, interval between the last dose and time of sample collection for the outcome assessment and average age of the participants on the outcome parameters using meta-regression analyses.

## 2. Methods

The study protocol was registered at PROSPERO (ID = CRD42018108202) [20] and executed as per the recommendations of “Preferred Reporting Items for Systematic reviews and Meta-analysis (PRISMA)” [21].

### 2.1. Criteria for Considering the Studies

Randomized and non-randomized controlled studies directly investigating the relative efficacy of ergocalciferol and cholecalciferol intervention (by either conventional supplementation/food fortification) in apparently healthy human participants were considered for the review. Studies explicitly intervening in patients with either acute or chronic conditions such as cardiovascular, liver, kidney, neuropsychiatric disorders, Human Immunodeficiency Virus (HIV) infection, cystic fibrosis and cancer were excluded.

### 2.2. Search Methods for Identification of Studies

Potential studies were identified by systematic search of various digital repositories (PubMed, Cochrane, Latin American and Caribbean Health Sciences Literature, Scientific Electronic Library Online, Pan American Health Library, WHO Library and Indian Medical Journals), clinical registries (WHO, European union, NIH U.S. National Library of Medicine, International Standard Randomized Controlled Trial Number Registry, Clinical Trials Registry-India, German Clinical Trials Register, Pan African Clinical Trial Registry, The Netherlands National Trial Register, Norway clinical research) and conference proceedings using key words (systematically searched sources are listed at PROSPERO registration ID = CRD42018108202) [20]. The search terms were constructed on the basis of the PICOS (i.e., participants, intervention, control, outcome and study type) strategy endorsed by Cochrane collaboration [21]. The details of electronic search terms and inclusion/exclusion criteria are provided at the PROSPERO registration [20] and in the Appendix. The electronic search was initially performed from the date of inception to 31 September 2019 and updated on 19 June 2021. We employed “sensitivity and precision maximizing version” strategy to identify the relevant studies [21].

### 2.3. Data Collection and Analysis

All citations resulting from the electronic search were compiled using Endnote (Version 9), and duplicates were removed. Authors (R.B., R.P.) independently screened titles and abstracts of all the articles for their inclusion.

Full texts of articles identified during screening were further scrutinized for their inclusion. Information on the estimates of vitamin D metabolites such as serum levels of total 25(OH)D, 25(OH)D_2_, 25(OH)D_3_, functional indicators such as serum PTH, isometric muscle strength, hand grip strength and bone mineral density were extracted from each of the included studies, wherever available.

### 2.4. Data Extraction and Management

A structured data sheet was used to extract details from the included studies such as the year of publication, country/place of study, details of the intervention (duration, dosage, route of administration, vehicle used for supplementation and season, interval between the last dose and the test for outcome assessment), sample size, male–female ratio, mean and standard deviation of outcome parameters (vitamin D metabolites and its functional markers described above) and techniques employed to measure the outcome parameters. Duplication of data (publication) was investigated in the included studies as recommended by Cochrane (Section 5) [21].

### 2.5. Assessment of Risk of Bias

The risk of bias was independently evaluated by the authors using a structured spread sheet. The domains–random sequence generation, allocation concealment, blinding of participants and personnel, blinding of outcome assessment, incomplete outcome data, selective reporting and other bias were rated according to the ‘Risk of bias’ assessment tool described in Cochrane Handbook for Systematic Reviews of Interventions [21]. Disagreement was resolved by discussion among authors (R.B., R.P. and B.K.). Additionally, funnel plot symmetry was visually inspected to assess publication bias as a source of heterogeneity.

### 2.6. Measures of Treatment Effect

Mean and standard deviation (SD) (or equivalent) of the outcome variables were pooled from all the included studies to execute the meta-analysis. Studies reporting post-intervention changes (Δ) from baseline were directly recorded for quantitative analysis [18,22,23,24,25,26,27,28]. In case of studies reporting baseline and final (post-intervention) values [16,19,29,30,31,32,33,34,35,36,37,38,39,40], the mean and SD of post-intervention changes were calculated using Monte Carlo simulation (Microsoft excel function). Lastly, for those studies reporting the results with box-and-whisker plots, a web-plot application was used to manually extract mean and confidence intervals [41]. The SD was derived from confidence interval using $SD=\frac{\sqrt{n}*\left(upperCI-lowerCI\right)}{2*\left(‘T\alpha ,df\right)}$ (where, SD, CI, Tα and df indicate standard deviation, confidence interval, t value distribution and degree of freedom respectively) [21]. All included studies analyzed and reported the results as per the principles of intention-to-treat.

Meta-analysis was conducted using RevMan 5.3 [42] utilizing the generic inverse variance method. A random-effect model was used in anticipation of contextual heterogeneity among the studies. Two-sided *p* < 0.05 was considered statistically significant.

### 2.7. Assessment of Heterogeneity

The influence of heterogeneity was evaluated by (1) visual inspection (inconsistency) of forest plots, (2) standard Chi^2^ test (*p* < 0.1) and (3) *I*^2^ statistic (>75%). Further, the source of heterogeneity was investigated by manually inspecting variables (sensitivity analysis) such as study participants, study setting, dose and duration of the intervention and co-interventions as well as methodological factors including study duration, season, method of sequence generation, allocation concealment, blinding of outcome assessment and losses to follow-up. Additionally, the heterogeneity due to study-level characteristics was explored using sub-group analyses and meta-regression with the random effect model.

### 2.8. Subgroup Analysis and Sensitivity Analysis

Subgroup analysis was performed when a minimum of 10 studies were available. We performed several subgroup analyses based on (a) baseline vitamin D status (serum 25(OH)D level <50 nmol/L vs. ≥50 nmol/L), (b) frequency of intervention (daily vs. single dose), (c) total intervention dose (<60,000 IU, 60,000–300,000 IU and >300,000 IU), (d) average dose per day (≤1000 IU, 1000–4000 IU and ≥ 4000 IU), (e) dose-test interval (≤14 day vs. >14 days), (f) age of the participants (<65 years vs. ≥65 years) and (g) analytical methods (radioimmunoassay (RIA)/high-performance liquid chromatography (HPLC)/liquid chromatography mass spectrometry (LCMS)). Two-sided *p* < 0.1 was considered statistically significant for the subgroup analysis and heterogeneity test [43].

### 2.9. Meta-Regression

Meta-regression was conducted to explore the contribution of various study characteristics on heterogeneity (for continuous variables). Bubble plots were constructed for those variables identified to be significant (*p* < 0.05). R-studio (Metafor package) was used for conducting meta-regression analysis.

## 3. Results

**Figure 1:**
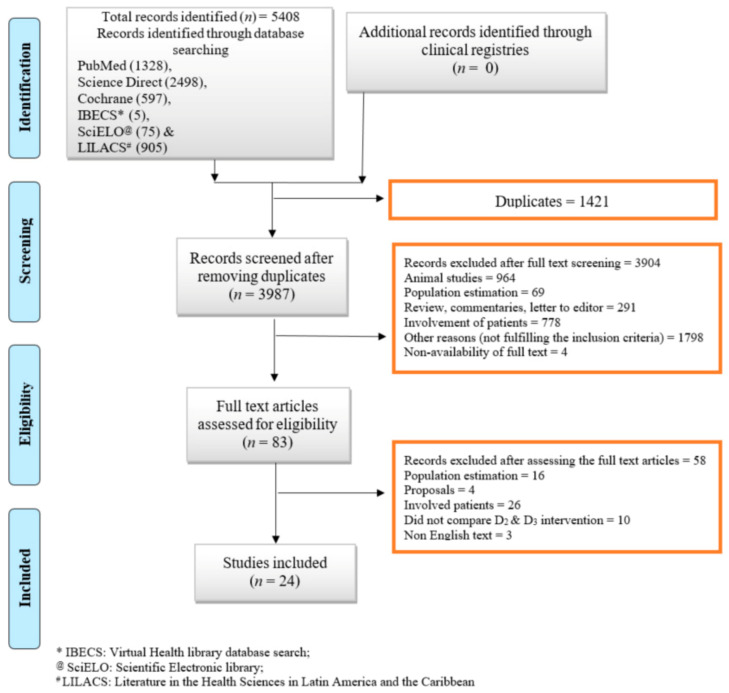
Flow diagram as per PRISMA recommendations. Legend: The flow chart illustrates the number of articles included and excluded at various steps.

The details of the electronic search and studies excluded at intermediate steps are described in the flowchart (Figure 1). The systematic review identified 24 studies; however, data from two studies were not included in the quantitative analysis (meta-analysis) because precise estimates of central tendency and data dispersion were not available due to graphical reporting and lack of response to our request from the primary authors [43,44]. Therefore, 22 studies were finally included in the quantitative data analysis (i.e., meta-analysis), whereas all 24 studies were included in the qualitative analysis (systematic review). All the studies meeting the inclusion and exclusion criteria involved random allocation of participants to receive either ergocalciferol or cholecalciferol (with occasionally an additional placebo group) for evaluating the efficacy of the two vitamers.

A total of 1277 participants were included in the meta-analysis, of which 644 received cholecalciferol and 633 received ergocalciferol intervention. Details regarding the study design, objectives, participants, interventions (as well as its adherence and dose received in the month before the outcome assessment), intake of additional calcium supplements (with equal doses in the two arms), exposure to UV-B radiation (e.g., sunlight), analytical methods and outcome variables evaluated are described in Table 1. Further, the risk of bias for each domain for all the included studies is described in Table 2.

Details on the outcome parameters evaluated are listed in Table 1. Serum total 25(OH)D was evaluated in all but one study. Ten studies measured 25(OH)D_3_ and 25(OH)D_2_ levels individually [16,23,25,27,32,33,34,35,39,45], whereas the remaining studies reported only total 25(OH)D values [17,18,19,22,24,26,28,29,31,36,37,38]. Serum PTH levels were reported by seven studies [16,17,18,25,27,29,36]. All the studies involved healthy individuals including elderly [18], postmenopausal women [18,32] and pre-pubertal children [28,44].

Except for studies Hammami et al. (2017) [43], Nimitphong et al. (2013) [27] and Thacher et al. (2010) [44], which were conducted at Saudi Arabia, Thailand and Nigeria, respectively, the rest of the studies were conducted in North America, Europe and Australian continents. None of the studies investigated functional outcomes such as muscle strength or bone density. Sheih et al. (2016) reported evaluating bone mineral density and muscle strength in their clinical trial registration (ClinicalTrials.gov Identifier: NCT01848236) [38]. However, the results on these outcomes are not available.

Risk of Bias: Risk of bias for the domains “random sequence generation”, “allocation concealment”, “blinding of participants and personnel”, “blinding of outcome assessment”, “incomplete outcome data”, “selective reporting” and other bias were rated as “low”, “unclear bias” and “high” risk of bias as described by Cochrane Handbook for Systematic Reviews of Interventions. None of the studies were biased by incomplete/selective reporting of outcome, while majority of studies had low risk of bias in terms of random selection of participants (random sequence generation) and blinding the participants and personnel (96% and 60%, respectively). However, majority of the studies did not provide clear description of the allocation concealment and blinding of outcome assessment (65.38% and 76.92%, respectively) (Table 2).

**Figure 2:**
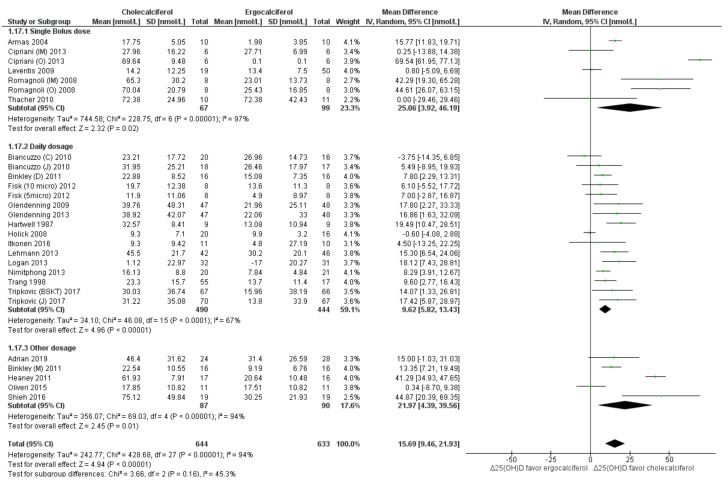
Forrest plot analysis of serum total 25(OH)D: sub-group analysis based on the frequency of doses. Legend: Forrest plot of random effect meta-analysis comparing the effects of cholecalciferol vs. ergocalciferol supplementation on net changes in 25(OH)D concentrations favored cholecalciferol. “Δ25(OH)D” denotes the change in total 25(OH)D concentrations from baseline (net change), “diamond” image denotes the mean differences (with 95% confidence interval). The pooled results indicate a mean difference of 15.69 nmol/L (95% confidence interval: 9.46–21.93 nmol/L) favoring cholecalciferol supplementation. Sub-group analyses in relation to the dosage frequency (single stat or bolus dose vs. daily dosage) are presented. Sub-group analyses show higher serum 25(OH)D levels among the cholecalciferol supplemented group as compared to ergocalciferol group. However, the studies are highly heterogeneous (*I*^2^ > 65%).

**Figure 3:**
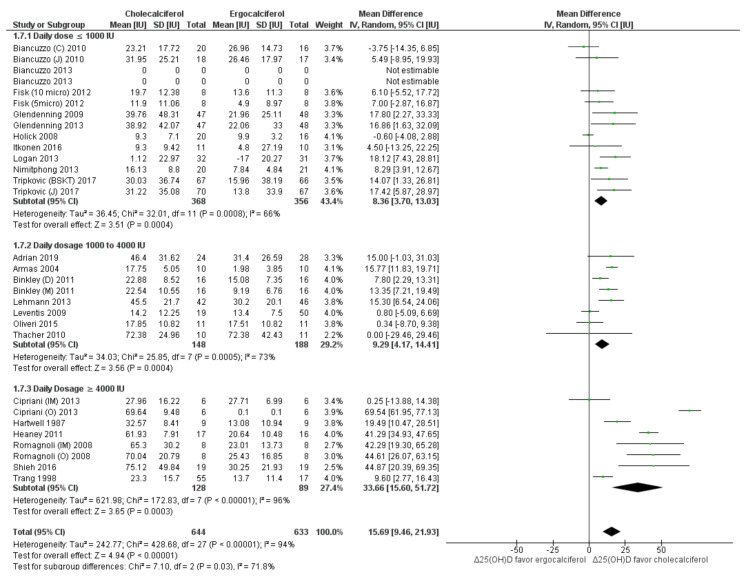
Forrest plot analysis of serum total 25(OH)D: sub-group analysis based on daily dosage. Legend: Forrest plot of random effect meta-analysis comparing the effects of cholecalciferol vs. ergocalciferol supplementation on net changes in 25(OH)D concentrations favored cholecalciferol. Sub-group analyses in studies with daily dosage ≤ 1000 nmol/L, 1000–4000 nmol/L and ≥ 4000 nmol/L of the respective vitamin D forms showed higher serum 25(OH)D levels among the cholecalciferol group as compared to ergocalciferol group in all groups. The heterogeneity of the subgroup analysis was high (*I*^2^ > 65%). The test of subgroup difference was statistically significant (*p* = 0.03).

**Figure 4:**
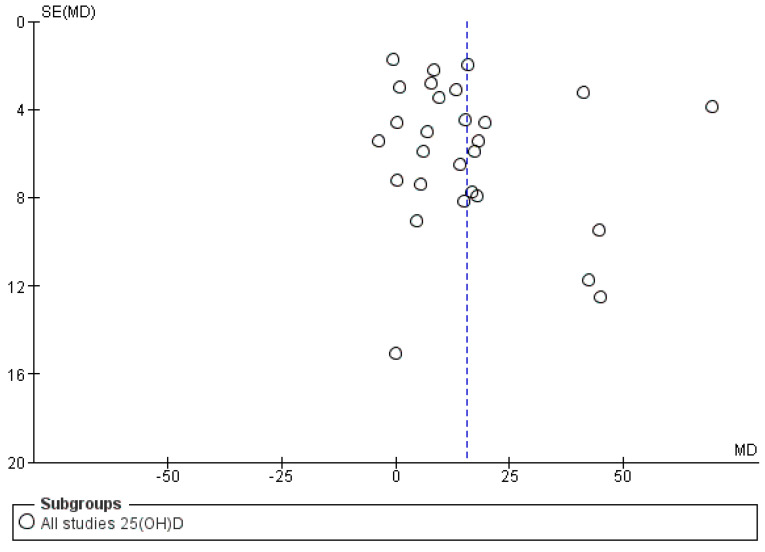
Funnel plot for total 25(OH)D. Legend: Funnel plot with X axis representing the estimated measure (standardized mean difference) of the 25(OH)D and Y axis representing the precision of the measure (standard error). The funnel plot suggests relatively mixed quality studies (due to variations in the standard error/SE).

Serum total 25(OH)D: On pooling results of the included studies, we found that cholecalciferol intervention elevated total 25(OH)D levels to a greater extent (*p* < 0.05) as compared to ergocalciferol (mean difference (MD): 15.69 nmol/L, 95% CI: 9.46 to 21.93) (Figure 2). However, the heterogeneity among the included studies was very high (*I*^2^ = 94%, *p* < 0.05). Sub-group analysis of studies with “daily intervention” protocol reduced heterogeneity (*I*^2^ = 67%) as well as the effect size (MD: 9.62 nmol/L, 95% CI: 5.82 to 13.43) when compared to the studies which provided a single bolus dose (MD: 25.06 nmol/L, 95% CI: 3.92 to 46.19) (Figure 2). Similarly, lower heterogeneity was observed in subgroups of studies which provided lower average daily intervention dose (≤1000 IU; *I*^2^ = 66%) (Figure 3), used HPLC and LCMS for outcome assessment (*I*^2^ = 9% and 64%, respectively) (Supplementary Figure S1) and had shorter dose-test interval (≤14 days; *I*^2^ = 67%) (Supplementary Figure S2). Sub-group of studies which provided total dose < 60,000 IU also had lower heterogeneity (*I*^2^ = 26%) (Supplementary Figure S3). On the other hand, analyses in subgroups of studies which used higher intervention doses (>1000 IU/day) (Figure 3), used RIA or other analytical methods (Supplementary Figure S1) and had longer dose-test interval (>14 days) (Supplementary Figure S2) and provided total dose > 60,000 IU (Supplementary Figure S3) had higher heterogeneity (*I*^2^ > 75%). Sub-group analyses in relation to participant age (<65 years vs. ≥65 years) (Supplementary Figure S4) and baseline vitamin D status (<50 nmol/L vs. ≥50 nmol/L) (Supplementary Figure S5) did not reduce heterogeneity. Visual inspection of the funnel plot to assess the source of heterogeneity attributable to publication bias was inconclusive (Figure 4).

The multivariate meta-regression analyses revealed that “average dose per day” was a significant predictor of effect size even after controlling for other study-level characteristics “mean age of the participants”, “total dose” and “dose-test interval” (Table 3 and Supplementary Figure S6).

**Figure 5:**
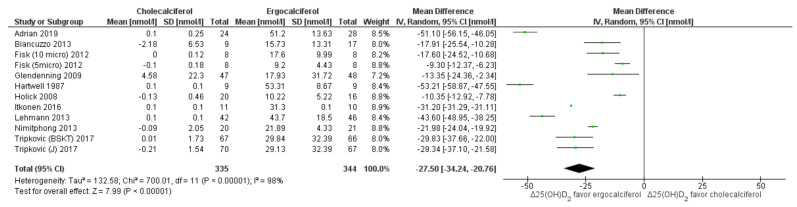
Forrest plot for 25(OH)D_2_. Legend: Forrest plot of random effect meta-analysis comparing the effects of cholecalciferol vs. ergocalciferol supplementation on net changes in 25(OH)D_2_ concentrations favored ergocalciferol. “Δ25(OH)D_2_” denotes the change in 25(OH)D_2_ concentrations from baseline (net change), squares denote the mean differences (with 95% confidence interval), i.e., the pooled results indicate mean difference of −27.5 nmol/L (95% confidence interval: −34.24 to −20.76 nmol/L), favoring ergocalciferol supplementation. However, the studies are highly heterogeneous (*I*^2^ = 98%).

**Figure 6:**
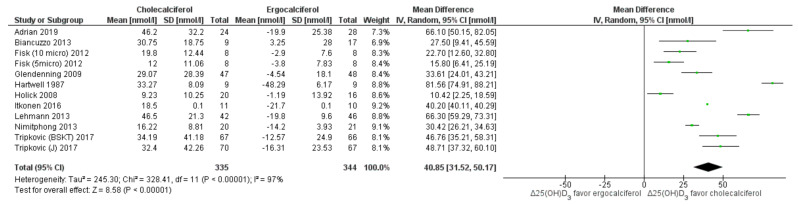
Forrest plot for 25(OH)D_3_**_._** Legend: Forrest plot of random effect meta-analysis comparing the effects of cholecalciferol vs. ergocalciferol supplementation on net changes in 25(OH)D_3_ concentrations favored cholecalciferol. “Δ25(OH)D_3_” denotes the change in 25(OH)D_3_ concentrations from baseline (net change), squares denote the mean differences (with 95% confidence interval), i.e., the pooled results indicate mean difference of 40.85 nmol/L with 95% confidence interval of 31.52 to 50.17 nmol/L, favoring cholecalciferol supplementation. However, the studies are highly (significantly) heterogeneous (*I*^2^ = 97%).

Serum levels of 25(OH)D_2_ and 25(OH)D_3_: Both cholecalciferol and ergocalciferol interventions favored greater increase in their respective 25(OH)D forms. Ergocalciferol intervention resulted in significantly higher 25(OH)D_2_ (MD: −27.5 nmol/L (95% CI: −34.24 to −20.76) (Figure 5), whereas cholecalciferol intervention elevated 25(OH)D_3_ to a significantly greater extent (MD: 40.85 nmol/L, 95% CI: 31.52 to 50.17, *p* < 0.05 nmol/L). However, the heterogeneity among the studies was very high (*I*^2^ ≥ 94%) (Figure 6). Subgroup analyses were not possible as fewer studies reported serum 25(OH)D_2_ and 25(OH)D_3_ levels. In multivariate meta-regression analysis, “total dose”, “average dose per day”, “participant age” and “dose-test interval” were not significant predictors of effect size (Table 3).

**Figure 7:**
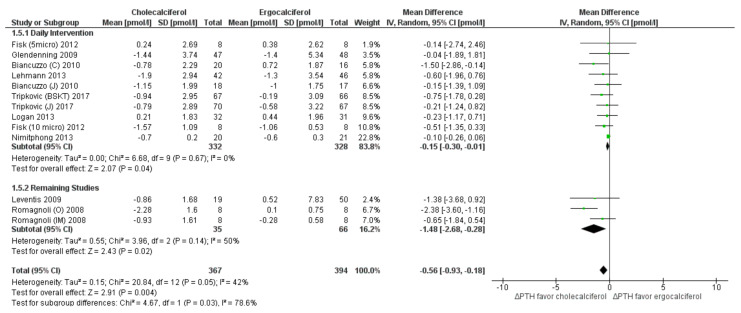
Forrest plot for parathyroid levels. Legend: Forrest plot of random effect meta-analysis comparing the effects of cholecalciferol vs. ergocalciferol supplementation on net changes in parathyroid (PTH) levels favored cholecalciferol. “ΔPTH” denotes the change in PTH concentrations from baseline (net change), squares denote the mean differences (with 95% confidence interval). The pooled results indicate a mean difference of 0.56 pmol/L (95% confidence interval 0.18–0.93 pmol/L), favoring cholecalciferol supplementation. Sub-group analyses in relation to dosage frequency (daily dosage vs. remaining studies) also demonstrated consistently higher PTH levels among the cholecalciferol-supplemented group as compared to the ergocalciferol group. However, the studies are moderately heterogeneous (*I*^2^ = 42%).

Parathyroid hormone: Although both ergocalciferol and cholecalciferol interventions promoted a fall in serum PTH levels, most studies documented larger reduction in the cholecalciferol group as compared to the ergocalciferol group. Meta-analysis suggested higher efficacy of cholecalciferol in reducing PTH levels than ergocalciferol (MD: −0.56 pmol/L; 95% CI: −0.93 to −0.18, *p* = 0.005) (Figure 7). There was moderate heterogeneity (*I*^2^ = 41%) within the included studies. Subgroup analysis in seven studies with daily intervention reduced heterogeneity (*I*^2^ = 0), as well as the magnitude of effect (MD = −0.15 pmol/L, 95% CI: −0.01 to −0.3, *p* = 0.04) (Figure 7). The meta-regression analyses showed that study-level characteristics “total dose”, “dose-test interval” “average dose per day” and “participant’s age” were not significant predictors of the effect size (Table 3).

## 4. Discussion

We analyzed the relative efficacy of ergocalciferol and cholecalciferol through a systematic review and meta-analysis, particularly focusing on different vitamin D metabolites (total 25(OH)D, 25(OH)D_2_ and 25(OH)D_3_) and a functional marker of calcium metabolism, PTH levels). Cholecalciferol supplementation was more efficacious than ergocalciferol in increasing total 25(OH)D levels and reducing PTH levels.

The qualitative analysis showed that, irrespective of the dosing frequency (single bolus/weekly/monthly/daily doses) or the mode or vehicle of administration (such as intramuscular injections, capsules, tablets, fortified orange juice, malt drink, biscuits or bread), cholecalciferol was more efficacious in raising serum total 25(OH)D levels. These results are in conformity with the earlier systematic review [15]. Our meta-analysis included fourteen randomized controlled trials in addition to the seven studies included in the previous meta-analysis. The mean difference in the Δ total 25(OH)D (15.69 nmol/L, 95%CI: 9.46 to 21.93) observed in our study was similar to the earlier review [15], suggesting this to be a stable estimate. None of the studies included in our review investigated functional outcomes. However, a previous systematic review has reported lower relative risk of mortality among those supplemented with cholecalciferol than those with ergocalciferol [46].

The studies included in the present meta-analysis were heterogeneous. The sub-group and meta-regression analyses conducted to explore the source of heterogeneity provide interesting insights. The sub-group analysis of studies which measured the outcome more than two weeks after the last dose of the intervention showed greater difference in Δ total 25(OH)D levels in the two groups (Supplementary Figure S2). Additionally, there was a greater difference in Δ total 25(OH)D at higher intervention doses and when the intervention was delivered as a bolus dose as against the daily doses (Figure 2 and Figure 3). These findings differ from the previous meta-analysis, which did not find significant difference in the impact of ergocalciferol and cholecalciferol in Δ total 25(OH)D when the data from 6 RCTs (*n* = 248 participants) implementing daily dosing were pooled. However, our analysis included 14 RCTs with daily dosing (*n* = 965 participants) and had higher statistical power. Greater difference in Δ total 25(OH)D levels in the two groups was also associated positively with average dose per day in the meta-regression analysis (Table 3 and Supplementary Figure S6).

Together, these findings suggest that the greater efficacy of cholecalciferol in raising serum levels of total 25(OH)D is likely with higher intervention doses, especially as bolus, and when the measurement is made more than two weeks after the last dose. The relatively lower potency of ergocalciferol in raising and maintaining total 25(OH)D could be attributed to the differences in structure (presence of additional methyl group at the 22nd carbon), its poor affinity to vitamin D binding protein leading to early degradation with shorter plasma half-life (13.9 days versus 15.1 days) [47].

Additionally, both ergocalciferol and cholecalciferol were relatively more beneficial in raising their respective forms of 25(OH)D compared to the other vitamer. As for Δ total 25(OH)D, the differences in the two groups were greater at higher intervention doses in case of Δ 25(OH)D_2_ and Δ 25(OH)D_3_. Interestingly, a few studies, but not all, have demonstrated decline in 25(OH)D_3_ after ergocalciferol supplementation indicating its degradation [32,35]. This could be linked with competition between ergocalciferol and cholecalciferol in binding 25-hydroxylase or vitamin D binding protein [48]. The studies included in the present meta-analysis, however, did not show a negative impact of cholecalciferol supplementation on 25(OH)D_2_.

Parathyroid hormone tightly regulates calcium homeostasis, at the expense of bone resorption; vitamin D induced regulation of PTH is therefore essential for bone health and integrity. The PTH suppression following vitamin D supplementation is due to the paracrine 1-hydroxylase in the parathyroid gland and other tissues [49]. There was a greater reduction in PTH levels with cholecalciferol, and meta-regression suggested lower difference at lower intervention doses. It is, however, noteworthy that majority of the included studies [17,25,27,29,31,35] did not report a significant difference in PTH (*p* > 0.05).

Our study has several strengths including comprehensive assessment of vitamin D metabolites as well as PTH, a larger sample size compared to previous meta-analysis, important sub-group analyses in relation to baseline vitamin D levels, intervention dose and frequency of dosing, analytical methods and dose-test interval, which provide important insights. Further, meta-regression analyses provide valuable information on predictors of the magnitude of difference between the impact of the two vitamers. However, the study has limitations that need to be acknowledged. The studies included in our systematic review were heterogeneous as some involved only women [18,32] or elderly [16,18,30,31]; participants with low vitamin D at baseline (<50 nmol/L); variable dosages and differing frequency of dosages such as single bolus or daily, weekly [26] or monthly doses [30]; and different methods (RIA, HPLC or LCMS) were used to estimate vitamin D metabolites. Bias assessment revealed that only two studies [25,39] provided clear description of methods and were deemed high quality, while the remaining studies had incomplete description of methods and were regarded as moderate quality. Moreover, the bulk of evidence in our meta-analysis is based on studies from the North America, Europe and Australia, with low representation of studies from the lower- and middle-income countries in Africa and South Asia, where dietary patterns and sun exposure are different and the results may not be generalizable. Studies in children and infants are also underrepresented in the current analysis.

## 5. Conclusions

The results suggest cholecalciferol to be more efficacious than ergocalciferol for increasing 25(OH)D levels and reducing serum PTH levels. However, both ergocalciferol and cholecalciferol interventions had higher efficacy in raising the serum levels of their respective forms of 25(OH)D (i.e., 25(OH)D_2_ and 25(OH)D_3_) when compared to the other vitamer. Cholecalciferol was more efficacious than ergocalciferol with bolus/intermittent doses, but frequent (daily) dosing was associated with lower differences for serum 25(OH)D and PTH levels. Thus, with lower doses typically used in fortified foods, cholecalciferol may be only marginally better than ergocalciferol for improving vitamin D status. Future studies evaluating the relative efficacy of ergocalciferol and cholecalciferol should also evaluate functional markers such as bone mineral density and muscle strength, and they should include longitudinal assessment at multiple time points to provide deeper insights on kinetics and dynamics of vitamin D. Lastly, studies from tropical areas, low- and middle-income country settings and younger populations (children and adolescents) are needed to understand the roles of nutrition and sun exposure in influencing the relative efficacy of the two vitamers.

## Figures and Tables

**Table 1:** Description of studies.

Study	Country	Participants	Duration of Follow up	Dosage and Duration of D_2_ and D_3_ Supplementation	Vitamin D Consumed in a Month before Outcome Assessment	Outcomes Assessed	Results
Adrian. 2019 [45]	United Kingdom (UK)	Apparently healthy adults with risk for diabetes between 30 and 75 years Cholecalciferol group: N = baseline 99, final 24 Ergocalciferol group: N = baseline 94, final 28	4 months	100,000 IU/month for 4 months, oral	400,000 IU	Total 25(OH)D, 25(OH)D_2_, 25(OH)D_3_, 1,25(OH)_2_D_2_, 1,25(OH)_2_D_3_	Ergocalciferol is less effective than cholecalciferol in elevating total 25(OH)D, and ergocalciferol reduces hydroxylation of vitamin D_3_ and 25(OH)D_3_.
Armas, 2004 [22]	United states of America (USA)	Apparently healthy men, age: range 20 to 61 years Cholecalciferol group: N = baseline 10, final 10 Ergocalciferol group: N = baseline 10, final 10	4 weeks	50,000 IU, single dose	50,000 IU	Total 25(OH)D	Cholecalciferol was found to be more potent with longer duration of action as compared to ergocalciferol.
Biancuzzo, 2013 [23]	United states of America (USA)	Apparently healthy adults, age: range 18 to 79 years Cholecalciferol group: N = baseline 9, final 9 (1 male) Ergocalciferol group: N = baseline 17, final 17 (7 males) Placebo: N = baseline 8, final 8 (1 male)	11 weeks	1000 IU/day for 11 weeks	30,000 IU	25(OH)D_2_ and 25(OH)D_3_	Ergocalciferol and cholecalciferol induced similar increases in total 25(OH)D as well as in 25(OH)D_2_ and 25(OH)D_3_, respectively.
Biancuzzo, 2010 [29]	United states of America (USA)	Apparently healthy adults, age 40.1 ± 15.6 years Cholecalciferol group (orange Juice): N = baseline 18, final 18 (3 males) Ergocalciferol group (orange Juice): N = baseline 17, final 17 (8 males) Apparently healthy adults, age 38.9 ± 12.3 years Cholecalciferol group (capsules): N = baseline 20, final 20 (8 males) Ergocalciferol group (capsules): N = baseline 16, final 16 (6 males)	11 weeks	1000 IU/day for 11 weeks	30,000 IU	Total 25(OH)D and PTH	Ergocalciferol and cholecalciferol were equally bioavailable in orange juice and capsules. D_2_ and cholecalciferol induced similar increases in total 25(OH)D as well as in 25(OH)D_2_ and 25(OH)D_3_, respectively.
Binkley, 2011 [30]	United states of America (USA)	Healthy older adults Cholecalciferol group (daily): N = baseline 16, final 16 (5 males), age: 74 ± 1.6 years Ergocalciferol group (daily): N = baseline 16, final 16 (7 males), age: 72.1 ± 1.9 years Cholecalciferol group (monthly): N = baseline 16, final 16 (6 males), age: 73.7 ± 1.4 years Ergocalciferol group(monthly): N = baseline 16, final 16 (5 males), age: 71.3 ± 1.4 years	1 year	1600 IU daily or 50,000 IU monthly for 1 year	48,000 IU or 50000 IU	Total 25(OH)D	Daily as well as monthly doses of cholecalciferol were marginally better than respective ergocalciferol doses in raising 25(OH)D.
Cipriani, 2013 [24]	Italy	Healthy adults, age 63.9 ± 7.1 years (18 females and 6 males) Cholecalciferol group: IM: N = baseline 6, final 6 Cholecalciferol group: Oral: N = baseline 6, final 6 Ergocalciferol group: IM: N = baseline 6, final 6 Ergocalciferol group: Oral: N = baseline 6, final 6	120 days	600,000 IU single dose (IM or oral)	Bolus dose received before 120 days, hence unavailable	Total 25(OH)D	Cholecalciferol was more effective than ergocalciferol in raising 25(OH)D and sustaining 1,25(OH)_2_D. Oral dosages produced immediate rise in active metabolites, while IM route provided slow but sustained increase in the metabolites.
Fisk, 2012 [25]	United Kingdom (UK)	Healthy adults 5 μg/day dosage: Cholecalciferol group: N = baseline 8, final 8 (3 males), age 30.5 ± 11 years 5 μg/day dosage: Ergocalciferol group: N = baseline 8, final 8 (3 males); age 24.4 ± 4.7 years 10 μg/day dosage: Cholecalciferol group: N = baseline 8, final 8 (5 males), age 30.6 ± 10.6 years; 10 μg/day dosage: Ergocalciferol group: N = baseline 8, final 8 (3 males), age 24.4 ± 3.9 years	4 weeks	200 IU/day or 400 IU/day through malted milk drink	56,000 IU or 11,200 IU	Total 25(OH)D, 25(OH)_2_, 25(OH)D_3_ and PTH	Both cholecalciferol and ergocalciferol resulted in dose-dependent increases in their respective 25(OH)D metabolites to a similar extent.
Glendenning, 2013 [31]	Australia	Adults with hip fracture and vitamin D insufficiency Cholecalciferol group: N = baseline 47, final 36, age 82 ±8 years Ergocalciferol group: N = baseline 48, final 34, age 84 ±9 years	3 months	1000 IU/day for 3 months	30,000 IU	Total 25(OH)D and Total 1,25(OH)_2_D	Compared to ergocalciferol, increment in total 25(OH)D was significantly higher with cholecalciferol, but there was no difference in total serum 1,25(OH)_2_D.
Glendenning, 2009 [16]	Australia	Adults with hip fracture and vitamin D insufficiency Cholecalciferol group: N = baseline 47, final 36, age 82 ± 8 years Ergocalciferol group: N = baseline 48, final 34, age 84 ± 9 years	3 months	1000 IU/day for 3 months	30,000 IU	Total 25(OH)D, 25(OH)D_2_, 25(OH)D_3_ and PTH	Compared to ergocalciferol, increment in total 25(OH)D was significantly higher with cholecalciferol, but there was no difference in the degree of PTH lowering between the treatments. Ergocalciferol and cholecalciferol supplementation.
Hammami, 2017 [43]	Saudi Arabia	Healthy adults Daily doses: Cholecalciferol group: N = baseline 34 (14 males), final 31, age 33.7 ± 9.7 years Daily doses: Ergocalciferol group: N = baseline 35 (14 males), final 28, age 34.7 ± 9.4 years Fortnightly doses: Cholecalciferol group: N = baseline 35 (14 males), final 30, age 33.4 ± 10.5 years Fortnightly doses: Ergocalciferol group: N = baseline 32 (14 males), final 26, age 31.5 ± 7.8 years 4 weekly doses: Cholecalciferol group: N = baseline 32 (14 males), final 25, age 31.4 ± 8.1 years 4 weekly doses: Ergocalciferol group: N = baseline 33 (13 males), final 27, age 33.5 ± 8 years	140 days	Daily 2000 IU/day or 25,000 IU fortnightly or 50,000 IU 4 weekly over 140 days	60,000 IU, 50,000 IU, 50,000 IU respectively	Total 25(OH)D, 25(OH)D_2_ and 25(OH)D_3_	Ergocalciferol had marginally higher efficacy than cholecalciferol in raising total 25(OH)D during the initial 14 days of daily supplementation. However, the latter was more efficacious with subsequent daily supplementation. Cholecalciferol was more efficacious in fortnightly and monthly supplementation.
Hartwell, 1987 [32]	Denmark	Premenopausal women, age 22 to 49 years Cholecalciferol group: N = baseline 9, final 9 Ergocalciferol group: N = baseline 9, final 9	8 weeks	4000 IU/day for 8 weeks	120,000 IU	Total 25(OH)D, 25(OH)D_2_and 25(OH)D_3_	Ergocalciferol intervention suppressed serum 1,25(OH)_2_D_3_ concentration while increasing 1,25(OH)_2_D_2._ The cholecalciferol intervention did not result in changes in 1,25(OH)_2_D metabolites.
Heaney, 2011 [26]	United states of America (USA)	Healthy adults Cholecalciferol group: N = baseline 17 (1 male), final 17, age: 49.3 ± 9.7 years Ergocalciferol group: N = baseline 16 (2 males), final 16, age: 49.7 ± 10.3 years	12 weeks	50,000 IU/week for 12 weeks	200,000 IU	Total 25(OH)D	Compared to ergocalciferol, cholecalciferol was found to be more potent in raising and maintaining total 25(OH)D levels.
Holick, 2008 [33]	United states of America (USA)	Healthy adults Cholecalciferol group: N = baseline 20 (7 males), final 20, age: 40 ± 18 years Ergocalciferol group: N = baseline 16 (6 males), final 16, age: 38.4 ± 12 years	11 weeks	1000 IU/day for 11 weeks	30,000 IU	Total 25(OH)D, 25(OH)D_2_and 25(OH)D_3_	Daily doses of both forms were equipotent in raising total 25(OH)D levels from their baseline value.
Itkonen, 2016 [34]	Finland	Healthy women Cholecalciferol group: N = baseline 11, final 11, age: 30.8 ± 3.7 years Ergocalciferol group: N = baseline 10, final 10, age: 25.6 ± 4.2 years	8 weeks	1000 IU/day for 8 weeks	30,000 IU	Total 25(OH)D, 25(OH)D_2_ and 25(OH)D_3_	Ergocalciferol was less potent than cholecalciferol in increasing the total 25(OH)D levels. Both ergocalciferol and cholecalciferol supplementation led to larger increases in their respective 25(OH)D metabolites than the other vitamer.
Lehmann, 2013 [35]	Germany	Healthy adults Cholecalciferol group: N = baseline 42 (16 males), final 35, age: 35.6 ± 13.5 years Ergocalciferol group: N = baseline 46 (15 males), final 42, age: 33.2 ± 12.4 years	8 weeks	2000 IU/day	60,000 IU	Total 25(OH)D, 25(OH)D_2_ and 25(OH)D_3_	Ergocalciferol was less potent than cholecalciferol in raising total 25(OH)D. Ergocalciferol supplementation was associated with a decrease in serum 25(OH)D_3_.
Leventis 2009 [17]	United Kingdom (UK)	Healthy adults Cholecalciferol group: N = baseline 19 (4 males), final 19, age: 43 (23–72) years Ergocalciferol group: N = baseline 50 (7 males), final 50, age: 53 (29−82 years)	24 weeks	D2: Single bolus 300,000 IU IM D3: 300,000 IU oral	Bolus dose received before 24 weeks, hence unavailable	Total 25(OH)D and PTH	Cholecalciferol had greater potency than ergocalciferol, with a higher, sustained serum 25(OH)D response and efficacious PTH suppression.
Logan, 2013 [36]	New Zealand	Healthy adults, age 18–50 years Cholecalciferol group: N = baseline 32, final 30 Ergocalciferol group: N = baseline 31, final 25	25 weeks	1000 IU/day for 25 weeks	30,000 IU	Total 25(OH)D and PTH	Cholecalciferol was more effective than ergocalciferol in raising total 25(OH)D levels, but no intervention-related changes in PTH were observed.
Nimitphong, 2013 [27]	Thailand	Healthy adults Cholecalciferol group: N = baseline 20 (3 males), final 20, age: 36 ± 1.9 years Ergocalciferol group: N = baseline 21 (4 males), final 19, age: 36.7 ± 1.7 years	3 months	400 IU/day for 3 months	12,000 IU	Total 25(OH)D, 25(OH)D_2_ 25(OH)D_3_ and PTH	Cholecalciferol-related increment in total 25(OH)D levels was higher than that with ergocalciferol. Genetic variations in DBP (rs4588 SNP) influenced 25(OH)D levels with cholecalciferol but not ergocalciferol.
Oliveri, 2015 [36]	Argentina	Healthy adults Cholecalciferol group: N = baseline 11 (3 males), final 11, age: 33.5 ± 7 years Ergocalciferol group: N = baseline 11 (2 males), final 11, age: 32.2 ± 5 years	77 days (values at the end of 21 days were considered, as values post 77 days were unavailable)	100,000 IU stat (day 0) + 4800 IU/day (7th–20th day)	196,000 IU	Total 25(OH)D	Cholecalciferol and ergocalciferol raised total 25(OH)D levels equally after the loading dose; however, the effect of the former was more sustained.
Romagnoli, 2008 [18]	Italy	Elderly women from nursing care facilities Cholecalciferol group: IM: N = baseline 8, final 8, age: 80 ± 10.1 years Cholecalciferol group: Oral: N = baseline 8, final 8, age: 78.5 ± 7.5 years Ergocalciferol group: IM: N = baseline 8, final 8, age: 79.4 ± 4.6 years Ergocalciferol group: Oral: N=baseline 8, final 8, age: 80.6 ± 5 years	60 days	300,000 IU single dose	Single bolus dose received before 60 days, hence unavailable	Total 25(OH)D and PTH	Cholecalciferol was more potent than ergocalciferol in raising total 25(OH)D levels.
Shieh, 2016 [38]	United states of America (USA)	Healthy adults Cholecalciferol group: N = baseline 19, final 19, age: 56.4 ± 19.6 years Ergocalciferol group: N = baseline 19, final 19, age: 50.2 ± 18.8 years	5 weeks	50,000 IU twice a week for 5 weeks	400,000 IU	Total 25(OH)D and 1,25(OH)_2_D	Cholecalciferol-related increase in total 25(OH)D was higher compared to ergocalciferol_._
Thacher, 2010 [44]	Nigeria	Healthy pre-pubertal children Cholecalciferol group: N = baseline 10 (5 males), final 10, age: 22–57 months Ergocalciferol group: N = baseline 11 (5 males), final 11, age: 19–59 months	14 days	50,000 IU stat	50,000 IU	Total 25(OH)D and 1,25(OH)_2_D	Cholecalciferol and ergocalciferol resulted in equal improvement in total 25(OH)D and 1,25(OH)_2_D levels in apparently healthy children.
Trang, 1998 [19]	Canada	Healthy adults Cholecalciferol group: N = baseline 55 (19 males), final 55, age: 38 ± 9 years Ergocalciferol group: N = baseline 17 (5 males), final 17, age: 38 ± 9 years	14 days	4000 IU/day	56,000 IU	Total 25(OH)D and 1,25(OH)_2_D	Cholecalciferol was more potent than ergocalciferol in raising total 25(OH)D levels.
Tripkovic, 2017 [39]	United Kingdom (UK)	Healthy adults Cholecalciferol group (Biscuits): N = baseline 67, final 67, age: 43.7 ± 12.8 years Ergocalciferol group (Biscuits): N = baseline 66, final 66, age:43.2 ± 13.2 years Cholecalciferol group (Juice): N = baseline 70, final 70, age: 43 ± 12.73 years Ergocalciferol group (Juice): N = baseline 67, final 67, age:44.3 ± 11.2 years	12 weeks	600 IU/day (Biscuits or juice)	18,000 IU	Total 25(OH)D and 1,25(OH)_2_D	Cholecalciferol was more potent than ergocalciferol in raising total 25(OH)D levels.

**Table 2:** Risk of bias assessment.

Study	Random Sequence Generation	Allocation Concealment	Blinding of Participants and Personnel	Blinding of Outcome Assessment	Incomplete Outcome Data	Selective Reporting	Other Sources of Bias
Adrian, 2019 [45]	Low risk of bias	Unclear risk of bias	Low risk of bias	Unclear risk of bias	Low risk of bias	Unclear risk of bias	None
Armas, 2004 [22]	Low risk of bias	Unclear risk of bias	Low risk of bias	Unclear risk of bias	Low risk of bias	Unclear risk of bias	None
Biancuzzo, 2013 [23]	Low risk of bias	Unclear risk of bias	Low risk of bias	Unclear risk of bias	Low risk of bias	Unclear risk of bias	None
Biancuzzo, 2010 [29]	Low risk of bias	Unclear risk of bias	Low risk of bias	Unclear risk of bias	Low risk of bias	Low risk of bias	None
Binkley, 2011 [30]	Low risk of bias	Unclear risk of bias	Low risk of bias	Unclear risk of bias	Low risk of bias	Low risk of bias	None
Cipriani, 2013 [24]	Low risk of bias	Unclear risk of bias	Unclear risk of bias	Low risk of bias	Low risk of bias	Unclear risk of bias	None
Fisk, 2012 [25]	Low risk of bias	Low risk of bias	Low risk of bias	Low risk of bias	Low risk of bias	Low risk of bias	None
Glendenning, 2013 [31]	Low risk of bias	Low risk of bias	Low risk of bias	Unclear risk of bias	Low risk of bias	Unclear risk of bias	None
Glendenning, 2009 [16]	Low risk of bias	Low risk of bias	Low risk of bias	Unclear risk of bias	Low risk of bias	Unclear risk of bias	None
Hammami, 2017 [43]	Low risk of bias	Unclear risk of bias	Unclear risk of bias	Low risk of bias	Low risk of bias	Low risk of bias	None
Hartwell, 1987 [32]	Low risk of bias	Low risk of bias	Unclear risk of bias	Unclear risk of bias	Low risk of bias	Unclear risk of bias	None
Heaney, 2011 [26]	Low risk of bias	Unclear risk of bias	Unclear risk of bias	Unclear risk of bias	Low risk of bias	Low risk of bias	None
Holick, 2008 [33]	Low risk of bias	Unclear risk of bias	Low risk of bias	Unclear risk of bias	Low risk of bias	Unclear risk of bias	None
Itkonen, 2016 [34]	Low risk of bias	Low risk of bias	Low risk of bias	Unclear risk of bias	Low risk of bias	Unclear risk of bias	None
Lehmann, 2013 [35]	Low risk of bias	Unclear risk of bias	Low risk of bias	Low risk of bias	Low risk of bias	Low risk of bias	None
Leventis 2009 [17]	Unclear risk of bias	Unclear risk of bias	Unclear risk of bias	Unclear risk of bias	Low risk of bias	Low risk of bias	None
Logan, 2013 [36]	Low risk of bias	Unclear risk of bias	Low risk of bias	Unclear risk of bias	Low risk of bias	Low risk of bias	None
Nimitphong, 2013 [27]	Low risk of bias	Low risk of bias	Unclear risk of bias	Unclear risk of bias	Low risk of bias	Unclear risk of bias	None
Oliveri, 2015 [37]	Low risk of bias	Low risk of bias	Unclear risk of bias	Low risk of bias	Low risk of bias	Unclear risk of bias	None
Romagnoli, 2008 [18]	Low risk of bias	Unclear risk of bias	Unclear risk of bias	Unclear risk of bias	Low risk of bias	Unclear risk of bias	None
Shieh, 2016 [38]	Low risk of bias	Unclear risk of bias	Unclear risk of bias	Unclear risk of bias	Low risk of bias	Low risk of bias	None
Thacher, 2010 [44]	Low risk of bias	Low risk of bias	Low risk of bias	Unclear risk of bias	Low risk of bias	Unclear risk of bias	None
Trang, 1998 [19]	Low risk of bias	Unclear risk of bias	Low risk of bias	Unclear risk of bias	Low risk of bias	Low risk of bias	None
Tripkovic, 2017 [39]	Low risk of bias	Low risk of bias	Low risk of bias	Low risk of bias	Low risk of bias	Low risk of bias	None

Legend/description of Table 2: Risk of bias for individual studies was assessed as recommended by Cochrane group.

**Table 3:** Summary of multivariate meta-regression analysis.

Explanatory Variable	Slope (β Coefficient)	95% CI of the Slope	*p* Value for Individual Predictors	*p* Value for Model	Proportion of Variation Explained by Model
**Serum total 25(OH)D nmol/L (N = 22 studies)**					
Total dose (per 100 IU)	−0.0002	−0.0043, 0.0038	0.9047	0.010	37.34%
Average dose/day (per 100 IU)	0.5122	0.1517, 0.8727	0.0054		
Dose-test interval (days)	−0.0113	−0.1571, 0.1344	0.8788		
Participant’s age (years)	0.2695	−0.0874, 0.6264	0.1389		
**Serum PTH pmol/L (N = 10 studies)**					
Total dose (per 100 IU)	0.0002	−0.0007, −0.0012	0.6027	0.0797	79.57%
Average dose/day (per 100 IU)	−0.0296	−0.0986, 0.0188	0.1826		
Dose-test interval (days)	−0.0072	−0.0242, 0.0098	0.4076		
Participant’s age (years)	0.0068	−0.0007, 0.0012	0.6027		
**Serum total 25(OH)D_3_ nmol/L (N = 10 studies)**					
Total dose (per 100 IU)	−0.0117	−0.1188, 0.0954	0.8305	0.0047	52.88%
Average dose/day (per 100 IU)	2.2053	−3.5824, 7.993	0.4552		
Dose-test interval (days)	1.1842	−12.7882, 15.1566	0.8681		
Participant’s age (years)	0.0778	−0.9051, 1.0607	0.8767		
**Serum total 25(OH)D_2_ nmol/L (N = 10 studies)**					
Total dose (per 100 IU)	−0.0022	−0.0688, 0.0645	0.9494	0.0003	62.71%
Average dose/day (per 100 IU)	−0.9128	−4.5182, 2.6926	0.6197		
Dose-test interval (days)	0.0209	−8.6496, 8.6914	0.9962		
Participant’s age (years)	0.2058	−0.4418, 0.8535	0.5333		

Legend/description for Table 3: Table presents the results of random-effect model (multivariate) meta-regression analyses investigating the relationship of “Mean age of the participants” (grand mean of both groups), “total dose (per 100 IU)” (sum of all doses received between baseline to final assessment), “average dose/day (per 100 IU)”(computed as the ratio of total dose and total study duration) and “Dose-Test Interval” (duration in days between the last intervention received and sample collection) with the outcome parameters (viz. the mean differences in 25(OH)D, PTH, 25(OH)D_3_ and 25(OH)D_2_) between the cholecalciferol and ergocalciferol groups.

## Supplementary Materials

The following are available online at https://www.mdpi.com/article/10.3390/nu13103328/s1, Figure S1: Forest Plot analysis of total 25(OH)D: subgroup analyses in relation to the method of estimation of 25(OH)D. Figure S2: Forrest Plot analysis of total 25(OH)D: dose test interval wise sub-group analysis. Figure S3: Forrest Plot analysis of 25(OH)D: total dose wise sub-group analysis. Figure S4: Forrest Plot analysis of 25(OH)D: age group wise sub-group analysis. Figure S5: Forrest Plot analysis of 25(OH)D: sub-group analysis based on baseline vitamin D status. Figure S6: Bubble plot demonstrating the association between “average dose per day” as and mean difference between the 2 interventions

## Abbreviations

Vitamin D_2_	Ergocalciferol
Vitamin D_3_	Cholecalciferol
PTH	Paratharmone/parathyroid hormone
25(OH)D	25 hydroxy vitamin D
25(OH)D_2_	25 hydroxy ergocalciferol
25(OH)D_3_	25 hydroxy cholecalciferol
1,25(OH)2D	1,25 dihydroxy vitamin D
1,25(OH)2D_2_	1,25 dihydroxy ergocalciferol
1,25(OH)2D_3_	1,25 dihydroxy cholecalciferol
nmol/L	nano moles per liter
pmol/L	pico moles per liter
SD	standard deviation
CI	confidence interval
MD	Mean difference
Δ	Change
IU	International units
RIA	Radioimmunoassay
HPLC	High performance liquid chromatography
LCMS	Liquid chromatography mass spectrometry
RCT	Randomized controlled trial
