# Supplementary material for: Relative Efficacy of Vitamin D2 and Vitamin D3 in Improving Vitamin D Status: Systematic Review and Meta-Analysis
Source: Nutrients. 2021 Sep 23;13(10):3328. doi: 10.3390/nu13103328 (PMC8538717; doi:10.3390/nu13103328)
Supplement: Supplementary file 1 [file nutrients-13-03328-s001.zip › nutrients-1374331-supplementary.pdf]

**Supplementary Figure S1 Forest Plot analysis of total 25(OH)D: sub group analyses in relation to the method of estimation of 25(OH)D**

Forrest plot of random effect meta-analysis comparing the effects of cholecalciferol vs ergocalciferol supplementation on net changes in 25(OH)D concentrations. "Δ25(OH)D" denotes the change in total 25(OH)D concentrations from baseline (net change), squares denote the mean differences (with 95% confidence interval). Sub-group analyses to explore the method of 25(OH)D estimation (RIA, HPLC, LCMS) revealed higher serum total 25(OH)D levels among the cholecalciferol group than ergocalciferol group irrespective of the method used for estimation of 25(OH)D. The heterogeneity was low in case of the studies estimating 25(OH)D using HPLC ($I^2$ = 9%) and high for other subgroups($I^2$>65%).

| | Cholecalciferol | | | Ergocalciferol | | | | Mean Difference |
|---|---|---|---|---|---|---|---|---|
| **Study or Subgroup** | **Mean** | **SD** | **Total** | **Mean** | **SD** | **Total** | **Weight** | **IV, Random, 95% CI** |
| **1.14.1 RIA** | | | | | | | | |
| Armas 2004 | 17.75 | 5.05 | 10 | 1.98 | 3.85 | 10 | 4.1% | 15.77 [11.83, 19.71] |
| Cipriani (IM) 2013 | 27.96 | 16.22 | 6 | 27.71 | 6.99 | 6 | 3.4% | 0.25 [-13.88, 14.38] |
| Cipriani (O) 2013 | 69.64 | 9.48 | 6 | 0.1 | 0.1 | 6 | 3.9% | 69.54 [61.95, 77.13] |
| Oliveri 2015 | 17.85 | 10.82 | 11 | 17.51 | 10.82 | 11 | 3.8% | 0.34 [-8.70, 9.38] |
| Romagnoli (IM) 2008 | 65.3 | 30.2 | 8 | 23.01 | 13.73 | 8 | 2.7% | 42.29 [19.30, 65.28] |
| Romagnoli (O) 2008 | 70.04 | 20.79 | 8 | 25.43 | 16.85 | 8 | 3.0% | 44.61 [26.07, 63.15] |
| Trang 1998 | 23.3 | 15.7 | 55 | 13.7 | 11.4 | 17 | 4.0% | 9.60 [2.77, 16.43] |
| **Subtotal (95% CI)** | | | **104** | | | **66** | **24.9%** | **25.60 [6.28, 44.91]** |
| Heterogeneity: Tau² = 635.49; Chi² = 212.63, df = 6 (P < 0.00001); I² = 97% | | | | | | | | |
| Test for overall effect: Z = 2.60 (P = 0.009) | | | | | | | | |
| **1.14.2 HPLC** | | | | | | | | |
| Binkley (D) 2011 | 22.88 | 8.52 | 16 | 15.08 | 7.35 | 16 | 4.0% | 7.80 [2.29, 13.31] |
| Binkley (M) 2011 | 22.54 | 10.55 | 16 | 9.19 | 6.76 | 16 | 4.0% | 13.35 [7.21, 19.49] |
| Glendenning 2009 | 39.76 | 48.31 | 47 | 21.96 | 25.11 | 48 | 3.3% | 17.80 [2.27, 33.33] |
| Glendenning 2013 | 38.92 | 42.07 | 47 | 22.06 | 33 | 48 | 3.3% | 16.86 [1.63, 32.09] |
| Hartwell 1987 | 32.57 | 8.41 | 9 | 13.08 | 10.94 | 9 | 3.8% | 19.49 [10.47, 28.51] |
| Tripkovic (BSKT) 2017 | 30.03 | 36.74 | 67 | 15.96 | 38.19 | 66 | 3.5% | 14.07 [1.33, 26.81] |
| Tripkovic (J) 2017 | 31.22 | 35.08 | 70 | 13.8 | 33.9 | 67 | 3.6% | 17.42 [5.87, 28.97] |
| **Subtotal (95% CI)** | | | **272** | | | **270** | **25.7%** | **13.24 [9.69, 16.79]** |
| Heterogeneity: Tau² = 2.26; Chi² = 6.62, df = 6 (P = 0.36); I² = 9% | | | | | | | | |
| Test for overall effect: Z = 7.31 (P < 0.00001) | | | | | | | | |
| **1.14.3 LCMS** | | | | | | | | |
| Adrian 2019 | 46.4 | 31.62 | 24 | 31.4 | 26.59 | 28 | 3.3% | 15.00 [-1.03, 31.03] |
| Biancuzzo (C) 2010 | 23.21 | 17.72 | 20 | 26.96 | 14.73 | 16 | 3.7% | -3.75 [-14.35, 6.85] |
| Biancuzzo (J) 2010 | 31.95 | 25.21 | 18 | 26.46 | 17.97 | 17 | 3.4% | 5.49 [-8.95, 19.93] |
| Fisk (10 micro) 2012 | 19.7 | 12.38 | 8 | 13.6 | 11.3 | 8 | 3.6% | 6.10 [-5.52, 17.72] |
| Fisk (5micro) 2012 | 11.9 | 11.06 | 8 | 4.9 | 8.97 | 8 | 3.8% | 7.00 [-2.87, 16.87] |
| Holick 2008 | 9.3 | 7.1 | 20 | 9.9 | 3.2 | 16 | 4.1% | -0.60 [-4.08, 2.88] |
| Itkonen 2016 | 9.3 | 9.42 | 11 | 4.8 | 27.19 | 10 | 3.1% | 4.50 [-13.25, 22.25] |
| Lehmann 2013 | 45.5 | 21.7 | 42 | 30.2 | 20.1 | 46 | 3.8% | 15.30 [6.54, 24.06] |
| Logan 2013 | 1.12 | 22.97 | 32 | -17 | 20.27 | 31 | 3.7% | 18.12 [7.43, 28.81] |
| Nimitphong 2013 | 16.13 | 8.8 | 20 | 7.84 | 4.84 | 21 | 4.1% | 8.29 [3.91, 12.67] |
| Thacher 2010 | 72.38 | 24.96 | 10 | 72.38 | 42.43 | 11 | 2.2% | 0.00 [-29.46, 29.46] |
| **Subtotal (95% CI)** | | | **213** | | | **212** | **38.8%** | **6.90 [2.22, 11.58]** |
| Heterogeneity: Tau² = 31.58; Chi² = 27.55, df = 10 (P = 0.002); I² = 64% | | | | | | | | |
| Test for overall effect: Z = 2.89 (P = 0.004) | | | | | | | | |
| **1.14.4 Others** | | | | | | | | |
| Heaney 2011 | 61.93 | 7.91 | 17 | 20.64 | 10.48 | 16 | 4.0% | 41.29 [34.93, 47.65] |
| Leventis 2009 | 14.2 | 12.25 | 19 | 13.4 | 7.5 | 50 | 4.0% | 0.80 [-5.09, 6.69] |
| Shieh 2016 | 75.12 | 49.84 | 19 | 30.25 | 21.93 | 19 | 2.5% | 44.87 [20.39, 69.35] |
| **Subtotal (95% CI)** | | | **55** | | | **85** | **10.5%** | **28.09 [-4.36, 60.54]** |
| Heterogeneity: Tau² = 769.09; Chi² = 87.79, df = 2 (P < 0.00001); I² = 98% | | | | | | | | |
| Test for overall effect: Z = 1.70 (P = 0.09) | | | | | | | | |
| **Total (95% CI)** | | | **644** | | | **633** | **100.0%** | **15.69 [9.46, 21.93]** |
| Heterogeneity: Tau² = 242.77; Chi² = 428.68, df = 27 (P < 0.00001); I² = 94% | | | | | | | | |
| Test for overall effect: Z = 4.94 (P < 0.00001) | | | | | | | | |
| Test for subgroup differences: Chi² = 7.67, df = 3 (P = 0.05), I² = 60.9% | | | | | | | | |

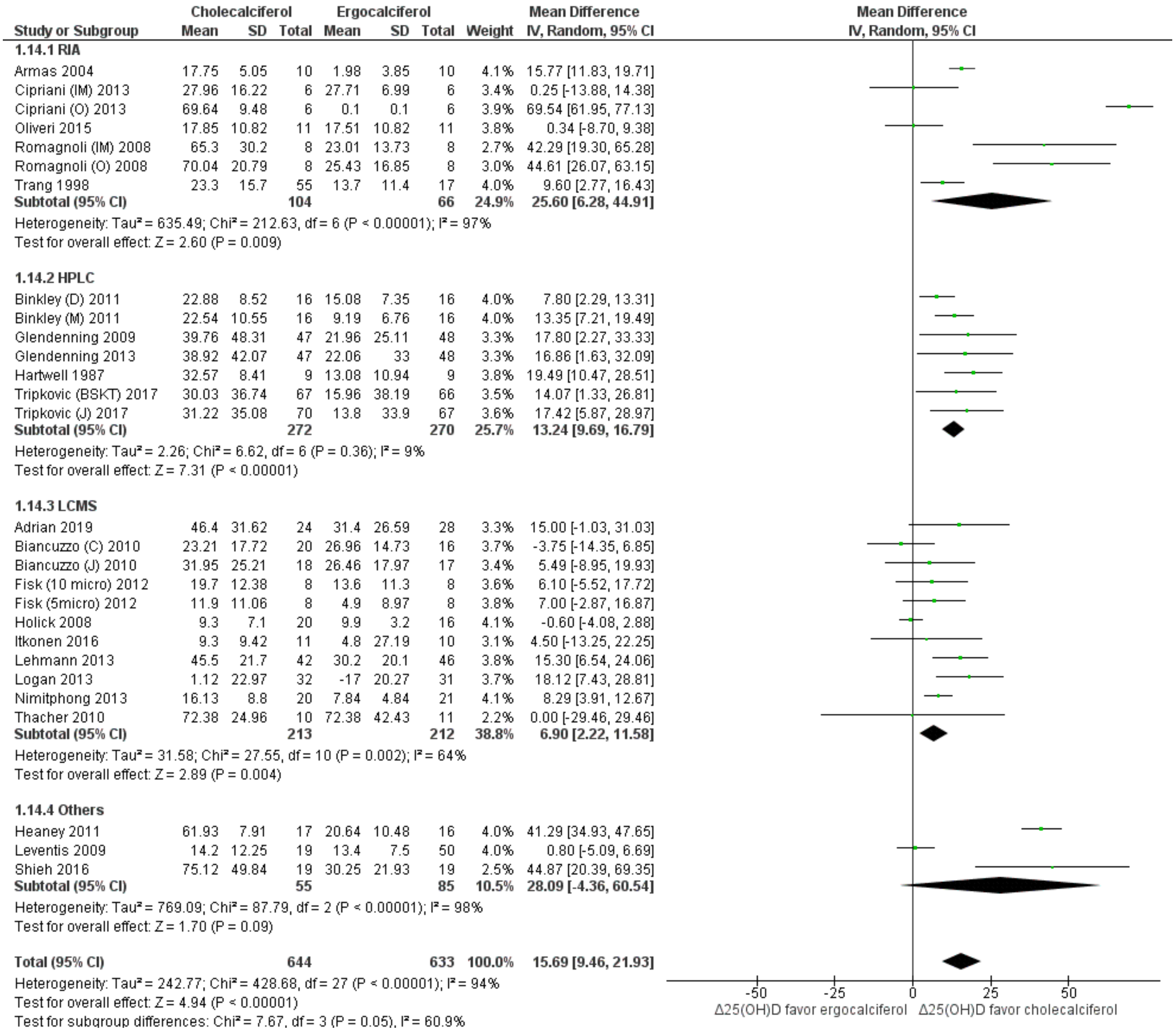

**Supplementary Figure S2**. **Forrest Plot analysis of total 25(OH)D: dose test interval wise sub-group analysis**

Forrest plot of random effect meta-analysis comparing the effects of cholecalciferol vs ergocalciferol supplementation on net changes in 25(OH)D concentrations. "Δ25(OH)D" denotes the change in total 25(OH)D concentrations from baseline (net change), squares denote the mean differences (with 95% confidence interval). Sub-group analyses in studies with different dose – test intervals (≤ 14 days *vs* > 14 days) revealed higher serum total 25(OH)D levels among the cholecalciferol supplemented group as compared to ergocalciferol group. The heterogeneity of the subgroup analysis was high ($I^2$> 67%). The test of subgroup difference was statistically significant.

| | Cholecalciferol | | | Ergocalciferol | | | | Mean Difference |
|---|---|---|---|---|---|---|---|---|
| **Study or Subgroup** | **Mean [nmol/l]** | **SD [nmol/l]** | **Total** | **Mean [nmol/l]** | **SD [nmol/l]** | **Total** | **Weight** | **IV, Random, 95% CI [nmol/l]** |
| **1.9.1 ≤ 14 days** | | | | | | | | |
| Biancuzzo (C) 2010 | 23.21 | 17.72 | 20 | 26.96 | 14.73 | 16 | 3.7% | -3.75 [-14.35, 6.85] |
| Biancuzzo (J) 2010 | 31.95 | 25.21 | 18 | 26.46 | 17.97 | 17 | 3.4% | 5.49 [-8.95, 19.93] |
| Fisk (10 micro) 2012 | 19.7 | 12.38 | 8 | 13.6 | 11.3 | 8 | 3.6% | 6.10 [-5.52, 17.72] |
| Fisk (5micro) 2012 | 11.9 | 11.06 | 8 | 4.9 | 8.97 | 8 | 3.8% | 7.00 [-2.87, 16.87] |
| Glendenning 2009 | 39.76 | 48.31 | 47 | 21.96 | 25.11 | 48 | 3.3% | 17.80 [2.27, 33.33] |
| Glendenning 2013 | 38.92 | 42.07 | 47 | 22.06 | 33 | 48 | 3.3% | 16.86 [1.63, 32.09] |
| Hartwell 1987 | 32.57 | 8.41 | 9 | 13.08 | 10.94 | 9 | 3.8% | 19.49 [10.47, 28.51] |
| Holick 2008 | 9.3 | 7.1 | 20 | 9.9 | 3.2 | 16 | 4.1% | -0.60 [-4.08, 2.88] |
| Itkonen 2016 | 9.3 | 9.42 | 11 | 4.8 | 27.19 | 10 | 3.1% | 4.50 [-13.25, 22.25] |
| Lehmann 2013 | 45.5 | 21.7 | 42 | 30.2 | 20.1 | 46 | 3.8% | 15.30 [6.54, 24.06] |
| Logan 2013 | 1.12 | 22.97 | 32 | -17 | 20.27 | 31 | 3.7% | 18.12 [7.43, 28.81] |
| Nimitphong 2013 | 16.13 | 8.8 | 20 | 7.84 | 4.84 | 21 | 4.1% | 8.29 [3.91, 12.67] |
| Oliveri 2015 | 17.85 | 10.82 | 11 | 17.51 | 10.82 | 11 | 3.8% | 0.34 [-8.70, 9.38] |
| Thacher 2010 | 72.38 | 24.96 | 10 | 72.38 | 42.43 | 11 | 2.2% | 0.00 [-29.46, 29.46] |
| Trang 1998 | 23.3 | 15.7 | 55 | 13.7 | 11.4 | 17 | 4.0% | 9.60 [2.77, 16.43] |
| Tripkovic (BSKT) 2017 | 30.03 | 36.74 | 67 | 15.96 | 38.19 | 66 | 3.5% | 14.07 [1.33, 26.81] |
| Tripkovic (J) 2017 | 31.22 | 35.08 | 70 | 13.8 | 33.9 | 67 | 3.6% | 17.42 [5.87, 28.97] |
| **Subtotal (95% CI)** | | | **495** | | | **450** | **61.0%** | **9.05 [5.10, 13.00]** |
| Heterogeneity: Tau² = 38.20; Chi² = 48.02, df = 16 (P < 0.0001); I² = 67% | | | | | | | | |
| Test for overall effect: Z = 4.49 (P < 0.00001) | | | | | | | | |
| **1.9.2 > 14 days** | | | | | | | | |
| Adrian 2019 | 46.4 | 31.62 | 24 | 31.4 | 26.59 | 28 | 3.3% | 15.00 [-1.03, 31.03] |
| Armas 2004 | 17.75 | 5.05 | 10 | 1.98 | 3.85 | 10 | 4.1% | 15.77 [11.83, 19.71] |
| Binkley (D) 2011 | 22.88 | 8.52 | 16 | 15.08 | 7.35 | 16 | 4.0% | 7.80 [2.29, 13.31] |
| Binkley (M) 2011 | 22.54 | 10.55 | 16 | 9.19 | 6.76 | 16 | 4.0% | 13.35 [7.21, 19.49] |
| Cipriani (IM) 2013 | 27.96 | 16.22 | 6 | 27.71 | 6.99 | 6 | 3.4% | 0.25 [-13.88, 14.38] |
| Cipriani (O) 2013 | 69.64 | 9.48 | 6 | 0.1 | 0.1 | 6 | 3.9% | 69.54 [61.95, 77.13] |
| Heaney 2011 | 61.93 | 7.91 | 17 | 20.64 | 10.48 | 16 | 4.0% | 41.29 [34.93, 47.65] |
| Leventis 2009 | 14.2 | 12.25 | 19 | 13.4 | 7.5 | 50 | 4.0% | 0.80 [-5.09, 6.69] |
| Romagnoli (IM) 2008 | 65.3 | 30.2 | 8 | 23.01 | 13.73 | 8 | 2.7% | 42.29 [19.30, 65.28] |
| Romagnoli (O) 2008 | 70.04 | 20.79 | 8 | 25.43 | 16.85 | 8 | 3.0% | 44.61 [26.07, 63.15] |
| Shieh 2016 | 75.12 | 49.84 | 19 | 30.25 | 21.93 | 19 | 2.5% | 44.87 [20.39, 69.35] |
| **Subtotal (95% CI)** | | | **149** | | | **183** | **39.0%** | **26.11 [13.20, 39.02]** |
| Heterogeneity: Tau² = 431.83; Chi² = 297.63, df = 10 (P < 0.00001); I² = 97% | | | | | | | | |
| Test for overall effect: Z = 3.96 (P < 0.0001) | | | | | | | | |
| **Total (95% CI)** | | | **644** | | | **633** | **100.0%** | **15.69 [9.46, 21.93]** |
| Heterogeneity: Tau² = 242.77; Chi² = 428.68, df = 27 (P < 0.00001); I² = 94% | | | | | | | | |
| Test for overall effect: Z = 4.94 (P < 0.00001) | | | | | | | | |
| Test for subgroup differences: Chi² = 6.13, df = 1 (P = 0.01), I² = 83.7% | | | | | | | | |

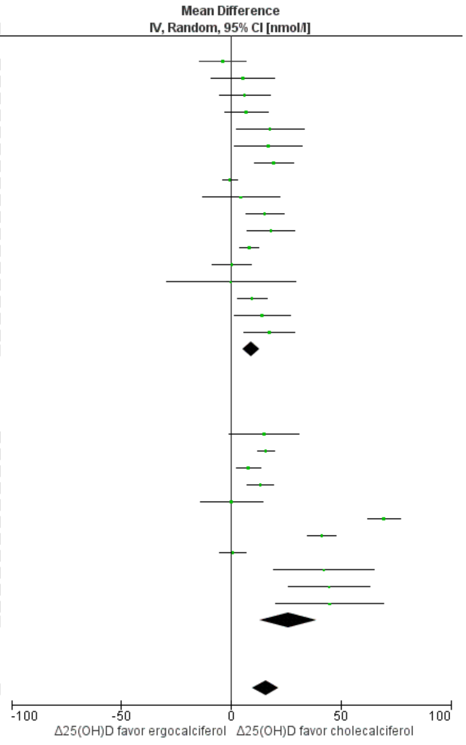

**Supplementary Figure S3**. **Forrest Plot analysis of 25(OH)D: total dose wise sub-group analysis**

Forrest plot of random effect meta-analysis comparing the effects of supplementing cholecalciferol as compared to ergocalciferol on net changes in 25(OH)D concentrations. "Δ25(OH)D" denotes the change in total 25(OH)D concentrations from baseline (net change), squares denote the mean differences (with 95% confidence interval). Sub-group analyses in studies with different categories of total dose (< 60,000 IU, 60000-300000 IU and > 300000 IU) revealed higher serum 25(OH)D levels among the cholecalciferol supplemented group as compared to ergocalciferol group The heterogeneity of the subgroup with total dose < 60,000 IU was low ($I^2$ =26 %). The test of subgroup difference was not statistically significant.

| | Cholecalciferol | | | Ergocalciferol | | | | Mean Difference |
|---|---|---|---|---|---|---|---|---|
| **Study or Subgroup** | **Mean** | **SD** | **Total** | **Mean** | **SD** | **Total** | **Weight** | **IV, Random, 95% CI** |
| **1.6.1 Total interventional Dose < 60,000 IU** | | | | | | | | |
| Armas 2004 | 17.75 | 5.05 | 10 | 1.98 | 3.85 | 10 | 4.1% | 15.77 [11.83, 19.71] |
| Fisk (10 micro) 2012 | 19.7 | 12.38 | 8 | 13.6 | 11.3 | 8 | 3.6% | 6.10 [-5.52, 17.72] |
| Fisk (5micro) 2012 | 11.9 | 11.06 | 8 | 4.9 | 8.97 | 8 | 3.8% | 7.00 [-2.87, 16.87] |
| Itkonen 2016 | 9.3 | 9.42 | 11 | 4.8 | 27.19 | 10 | 3.1% | 4.50 [-13.25, 22.25] |
| Nimitphong 2013 | 16.13 | 8.8 | 20 | 7.84 | 4.84 | 21 | 4.1% | 8.29 [3.91, 12.67] |
| Thacher 2010 | 72.38 | 24.96 | 10 | 72.38 | 42.43 | 11 | 2.2% | 0.00 [-29.46, 29.46] |
| Trang 1998 | 23.3 | 15.7 | 55 | 13.7 | 11.4 | 17 | 4.0% | 9.60 [2.77, 16.43] |
| Tripkovic (BSKT) 2017 | 30.03 | 36.74 | 67 | 15.96 | 38.19 | 66 | 3.5% | 14.07 [1.33, 26.81] |
| Tripkovic (J) 2017 | 31.22 | 35.08 | 70 | 13.8 | 33.9 | 67 | 3.6% | 17.42 [5.87, 28.97] |
| **Subtotal (95% CI)** | | | **259** | | | **218** | **32.0%** | **11.03 [7.80, 14.25]** |
| Heterogeneity: Tau² = 5.73; Chi² = 10.86, df = 8 (P = 0.21); I² = 26% | | | | | | | | |
| Test for overall effect: Z = 6.70 (P < 0.00001) | | | | | | | | |
| **1.6.2 Total interventional dose 60,000 - 300,000 IU** | | | | | | | | |
| Biancuzzo (C) 2010 | 23.21 | 17.72 | 20 | 26.96 | 14.73 | 16 | 3.7% | -3.75 [-14.35, 6.85] |
| Biancuzzo (J) 2010 | 31.95 | 25.21 | 18 | 26.46 | 17.97 | 17 | 3.4% | 5.49 [-8.95, 19.93] |
| Glendenning 2009 | 39.76 | 48.31 | 47 | 21.96 | 25.11 | 48 | 3.3% | 17.80 [2.27, 33.33] |
| Glendenning 2013 | 38.92 | 42.07 | 47 | 22.06 | 33 | 48 | 3.3% | 16.86 [1.63, 32.09] |
| Hartwell 1987 | 32.57 | 8.41 | 9 | 13.08 | 10.94 | 9 | 3.8% | 19.49 [10.47, 28.51] |
| Holick 2008 | 9.3 | 7.1 | 20 | 9.9 | 3.2 | 16 | 4.1% | -0.60 [-4.08, 2.88] |
| Lehmann 2013 | 45.5 | 21.7 | 42 | 30.2 | 20.1 | 46 | 3.8% | 15.30 [6.54, 24.06] |
| Logan 2013 | 1.12 | 22.97 | 32 | -17 | 20.27 | 31 | 3.7% | 18.12 [7.43, 28.81] |
| Oliveri 2015 | 17.85 | 10.82 | 11 | 17.51 | 10.82 | 11 | 3.8% | 0.34 [-8.70, 9.38] |
| **Subtotal (95% CI)** | | | **246** | | | **242** | **33.1%** | **9.29 [2.41, 16.18]** |
| Heterogeneity: Tau² = 80.75; Chi² = 39.42, df = 8 (P < 0.00001); I² = 80% | | | | | | | | |
| Test for overall effect: Z = 2.65 (P = 0.008) | | | | | | | | |
| **1.6.3 Total Interventional Dose ≥ 300,000 IU** | | | | | | | | |
| Adrian 2019 | 46.4 | 31.62 | 24 | 31.4 | 26.59 | 28 | 3.3% | 15.00 [-1.03, 31.03] |
| Binkley (D) 2011 | 22.88 | 8.52 | 16 | 15.08 | 7.35 | 16 | 4.0% | 7.80 [2.29, 13.31] |
| Binkley (M) 2011 | 22.54 | 10.55 | 16 | 9.19 | 6.76 | 16 | 4.0% | 13.35 [7.21, 19.49] |
| Cipriani (IM) 2013 | 27.96 | 16.22 | 6 | 27.71 | 6.99 | 6 | 3.4% | 0.25 [-13.88, 14.38] |
| Cipriani (O) 2013 | 69.64 | 9.48 | 6 | 0.1 | 0.1 | 6 | 3.9% | 69.54 [61.95, 77.13] |
| Heaney 2011 | 61.93 | 7.91 | 17 | 20.64 | 10.48 | 16 | 4.0% | 41.29 [34.93, 47.65] |
| Leventis 2009 | 14.2 | 12.25 | 19 | 13.4 | 7.5 | 50 | 4.0% | 0.80 [-5.09, 6.69] |
| Romagnoli (IM) 2008 | 65.3 | 30.2 | 8 | 23.01 | 13.73 | 8 | 2.7% | 42.29 [19.30, 65.28] |
| Romagnoli (O) 2008 | 70.04 | 20.79 | 8 | 25.43 | 16.85 | 8 | 3.0% | 44.61 [26.07, 63.15] |
| Shieh 2016 | 75.12 | 49.84 | 19 | 30.25 | 21.93 | 19 | 2.5% | 44.87 [20.39, 69.35] |
| **Subtotal (95% CI)** | | | **139** | | | **173** | **34.9%** | **27.43 [11.53, 43.32]** |
| Heterogeneity: Tau² = 606.09; Chi² = 290.96, df = 9 (P < 0.00001); I² = 97% | | | | | | | | |
| Test for overall effect: Z = 3.38 (P = 0.0007) | | | | | | | | |
| **Total (95% CI)** | | | **644** | | | **633** | **100.0%** | **15.69 [9.46, 21.93]** |
| Heterogeneity: Tau² = 242.77; Chi² = 428.68, df = 27 (P < 0.00001); I² = 94% | | | | | | | | |
| Test for overall effect: Z = 4.94 (P < 0.00001) | | | | | | | | |
| Test for subgroup differences: Chi² = 4.31, df = 2 (P = 0.12), I² = 53.6% | | | | | | | | |

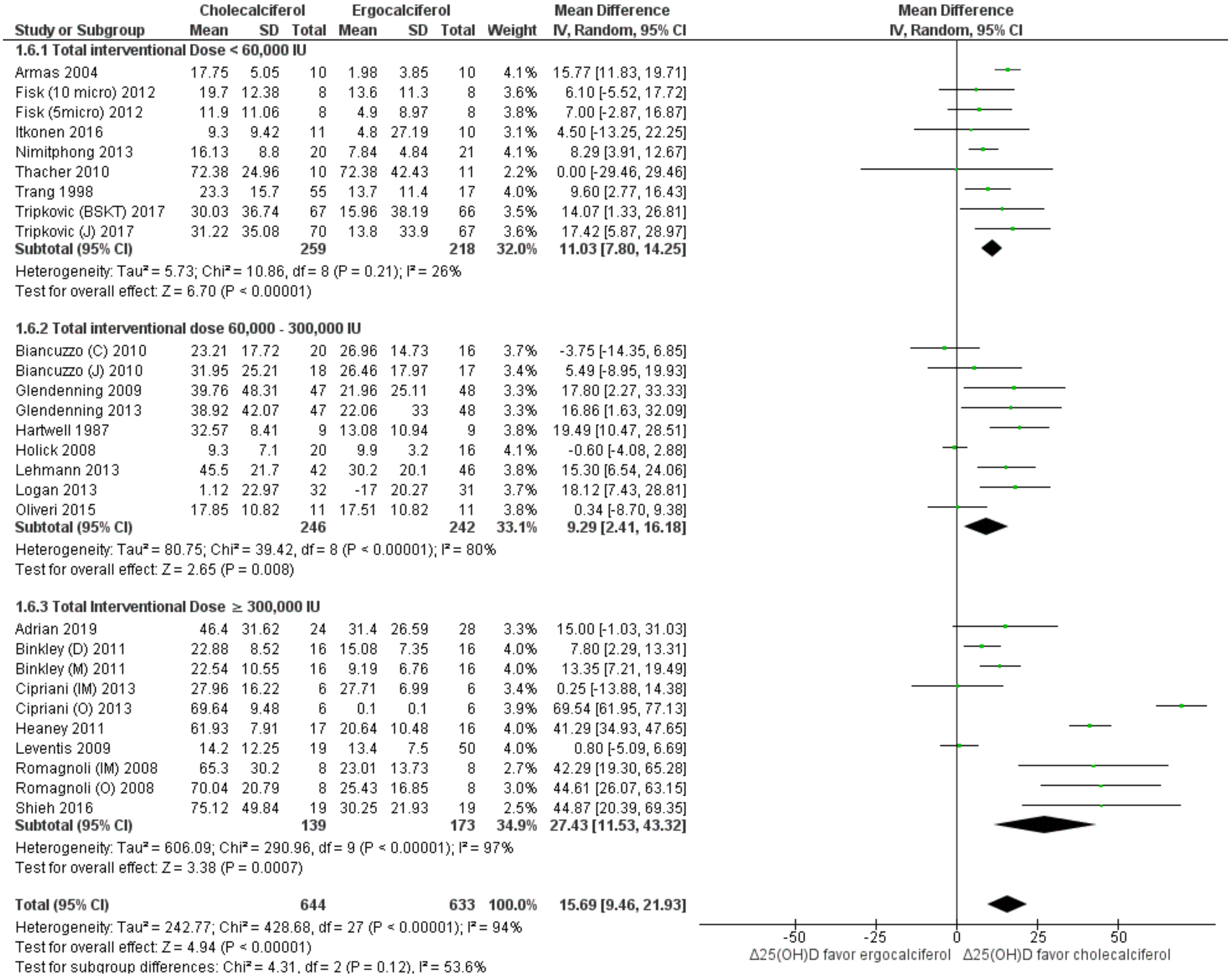

**Supplementary Figure S4. Forrest Plot analysis of 25(OH)D: age group wise sub-group analysis**

Forrest plot of random effect meta-analysis comparing the effects of supplementing cholecalciferol as compared to ergocalciferol on net changes in 25(OH)D concentrations. "Δ25(OH)D" denotes the change in total 25(OH)D concentrations from baseline (net change), squares denote the mean differences (with 95% confidence interval). Sub-group analyses in studies with different participant age groups (< 65 years, > 65 years and combined age groups) revealed higher serum 25(OH)D levels among the cholecalciferol supplemented group as compared to ergocalciferol group in studies that included participants < 65 years and > 65 years as compared to the studies which included both the age groups. The heterogeneity of the subgroup analysis was high ($I^2$> 75%). The test of subgroup difference was statistically insignificant.

| | Cholecalciferol | | | Ergocalciferol | | | | Mean Difference |
|---|---|---|---|---|---|---|---|---|
| **Study or Subgroup** | **Mean** | **SD** | **Total** | **Mean** | **SD** | **Total** | **Weight** | **IV, Random, 95% CI** |
| **1.15.1 Participants with < 65 years** | | | | | | | | |
| Armas 2004 | 17.75 | 5.05 | 10 | 1.98 | 3.85 | 10 | 4.1% | 15.77 [11.83, 19.71] |
| Fisk (10 micro) 2012 | 19.7 | 12.38 | 8 | 13.6 | 11.3 | 8 | 3.6% | 6.10 [-5.52, 17.72] |
| Fisk (5micro) 2012 | 11.9 | 11.06 | 8 | 4.9 | 8.97 | 8 | 3.8% | 7.00 [-2.87, 16.87] |
| Hartwell 1987 | 32.57 | 8.41 | 9 | 13.08 | 10.94 | 9 | 3.8% | 19.49 [10.47, 28.51] |
| Heaney 2011 | 61.93 | 7.91 | 17 | 20.64 | 10.48 | 16 | 4.0% | 41.29 [34.93, 47.65] |
| Itkonen 2016 | 9.3 | 9.42 | 11 | 4.8 | 27.19 | 10 | 3.1% | 4.50 [-13.25, 22.25] |
| Logan 2013 | 1.12 | 22.97 | 32 | -17 | 20.27 | 31 | 3.7% | 18.12 [7.43, 28.81] |
| Oliveri 2015 | 17.85 | 10.82 | 11 | 17.51 | 10.82 | 11 | 3.8% | 0.34 [-8.70, 9.38] |
| Shieh 2016 | 75.12 | 49.84 | 19 | 30.25 | 21.93 | 19 | 2.5% | 44.87 [20.39, 69.35] |
| Thacher 2010 | 72.38 | 24.96 | 10 | 72.38 | 42.43 | 11 | 2.2% | 0.00 [-29.46, 29.46] |
| Trang 1998 | 23.3 | 15.7 | 55 | 13.7 | 11.4 | 17 | 4.0% | 9.60 [2.77, 16.43] |
| Tripkovic (BSKT) 2017 | 30.03 | 36.74 | 67 | 15.96 | 38.19 | 66 | 3.5% | 14.07 [1.33, 26.81] |
| Tripkovic (J) 2017 | 31.22 | 35.08 | 70 | 13.8 | 33.9 | 67 | 3.6% | 17.42 [5.87, 28.97] |
| **Subtotal (95% CI)** | | | **327** | | | **283** | **45.8%** | **15.31 [8.09, 22.53]** |
| Heterogeneity: Tau² = 136.23; Chi² = 89.91, df = 12 (P < 0.00001); I² = 87% | | | | | | | | |
| Test for overall effect: Z = 4.15 (P < 0.0001) | | | | | | | | |
| **1.15.2 Participants with > 65 years** | | | | | | | | |
| Binkley (D) 2011 | 22.88 | 8.52 | 16 | 15.08 | 7.35 | 16 | 4.0% | 7.80 [2.29, 13.31] |
| Binkley (M) 2011 | 22.54 | 10.55 | 16 | 9.19 | 6.76 | 16 | 4.0% | 13.35 [7.21, 19.49] |
| Glendenning 2009 | 39.76 | 48.31 | 47 | 21.96 | 25.11 | 48 | 3.3% | 17.80 [2.27, 33.33] |
| Glendenning 2013 | 38.92 | 42.07 | 47 | 22.06 | 33 | 48 | 3.3% | 16.86 [1.63, 32.09] |
| Romagnoli (IM) 2008 | 65.3 | 30.2 | 8 | 23.01 | 13.73 | 8 | 2.7% | 42.29 [19.30, 65.28] |
| Romagnoli (O) 2008 | 70.04 | 20.79 | 8 | 25.43 | 16.85 | 8 | 3.0% | 44.61 [26.07, 63.15] |
| **Subtotal (95% CI)** | | | **142** | | | **144** | **20.4%** | **20.20 [10.79, 29.61]** |
| Heterogeneity: Tau² = 90.05; Chi² = 21.42, df = 5 (P = 0.0007); I² = 77% | | | | | | | | |
| Test for overall effect: Z = 4.21 (P < 0.0001) | | | | | | | | |
| **1.15.3 Studies involving participants with both age groups** | | | | | | | | |
| Adrian 2019 | 46.4 | 31.62 | 24 | 31.4 | 26.59 | 28 | 3.3% | 15.00 [-1.03, 31.03] |
| Biancuzzo (C) 2010 | 23.21 | 17.72 | 20 | 26.96 | 14.73 | 16 | 3.7% | -3.75 [-14.35, 6.85] |
| Biancuzzo (J) 2010 | 31.95 | 25.21 | 18 | 26.46 | 17.97 | 17 | 3.4% | 5.49 [-8.95, 19.93] |
| Cipriani (IM) 2013 | 27.96 | 16.22 | 6 | 27.71 | 6.99 | 6 | 3.4% | 0.25 [-13.88, 14.38] |
| Cipriani (O) 2013 | 69.64 | 9.48 | 6 | 0.1 | 0.1 | 6 | 3.9% | 69.54 [61.95, 77.13] |
| Holick 2008 | 9.3 | 7.1 | 20 | 9.9 | 3.2 | 16 | 4.1% | -0.60 [-4.08, 2.88] |
| Lehmann 2013 | 45.5 | 21.7 | 42 | 30.2 | 20.1 | 46 | 3.8% | 15.30 [6.54, 24.06] |
| Leventis 2009 | 14.2 | 12.25 | 19 | 13.4 | 7.5 | 50 | 4.0% | 0.80 [-5.09, 6.69] |
| Nimitphong 2013 | 16.13 | 8.8 | 20 | 7.84 | 4.84 | 21 | 4.1% | 8.29 [3.91, 12.67] |
| **Subtotal (95% CI)** | | | **175** | | | **206** | **33.8%** | **12.37 [-1.82, 26.56]** |
| Heterogeneity: Tau² = 444.47; Chi² = 291.04, df = 8 (P < 0.00001); I² = 97% | | | | | | | | |
| Test for overall effect: Z = 1.71 (P = 0.09) | | | | | | | | |
| **Total (95% CI)** | | | **644** | | | **633** | **100.0%** | **15.69 [9.46, 21.93]** |
| Heterogeneity: Tau² = 242.77; Chi² = 428.68, df = 27 (P < 0.00001); I² = 94% | | | | | | | | |
| Test for overall effect: Z = 4.94 (P < 0.00001) | | | | | | | | |
| Test for subgroup differences: Chi² = 1.02, df = 2 (P = 0.60), I² = 0% | | | | | | | | |

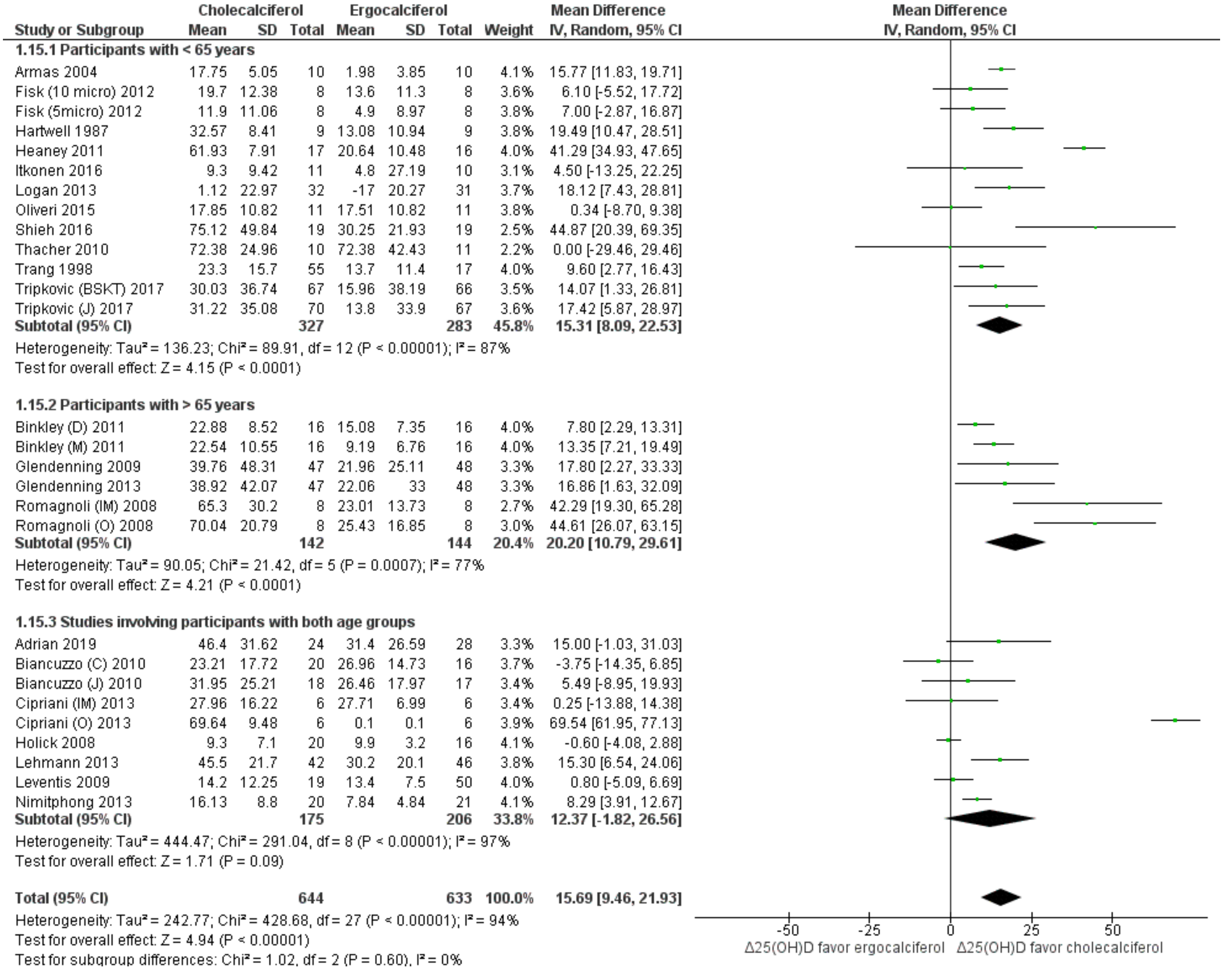

**Supplementary Figure S5. Forrest Plot analysis of 25(OH)D: sub-group analysis based on baseline vitamin D status**

Forrest plot of random effect meta-analysis comparing the effects of supplementing cholecalciferol as compared to ergocalciferol on net changes in 25(OH)D concentrations. "Δ25(OH)D" denotes the change in total 25(OH)D concentrations from baseline (net change). Sub-group analyses in studies with different baseline vitamin D levels in the participants (i.e. < 50 nmol/L *vs* > 50 nmol/L *vs* combined) revealed higher serum 25(OH)D levels among the cholecalciferol supplemented group as compared to ergocalciferol group The heterogeneity of the subgroup analysis was high ($I^2$> 80%). The test of subgroup difference was statistically insignificant.

| | Cholecalciferol | | | Ergocalciferol | | | | Mean Difference |
|---|---|---|---|---|---|---|---|---|
| Study or Subgroup | Mean [nmol/L] | SD [nmol/L] | Total | Mean [nmol/L] | SD [nmol/L] | Total | Weight | IV, Random, 95% CI [nmol/L] |
| **1.16.1 Baseline 25(OH)D < 50nmol / L** | | | | | | | | |
| Adrian 2019 | 46.4 | 31.62 | 24 | 31.4 | 26.59 | 28 | 3.3% | 15.00 [-1.03, 31.03] |
| Armas 2004 | 17.75 | 5.05 | 10 | 1.98 | 3.85 | 10 | 4.1% | 15.77 [11.83, 19.71] |
| Biancuzzo (C) 2010 | 23.21 | 17.72 | 20 | 26.96 | 14.73 | 16 | 3.7% | -3.75 [-14.35, 6.85] |
| Biancuzzo (J) 2010 | 31.95 | 25.21 | 18 | 26.46 | 17.97 | 17 | 3.4% | 5.49 [-8.95, 19.93] |
| Fisk (10 micro) 2012 | 19.7 | 12.38 | 8 | 13.6 | 11.3 | 8 | 3.6% | 6.10 [-5.52, 17.72] |
| Fisk (5micro) 2012 | 11.9 | 11.06 | 8 | 4.9 | 8.97 | 8 | 3.8% | 7.00 [-2.87, 16.87] |
| Glendenning 2009 | 39.76 | 48.31 | 47 | 21.96 | 25.11 | 48 | 3.3% | 17.80 [2.27, 33.33] |
| Glendenning 2013 | 38.92 | 42.07 | 47 | 22.06 | 33 | 48 | 3.3% | 16.86 [1.63, 32.09] |
| Holick 2008 | 9.3 | 7.1 | 20 | 9.9 | 3.2 | 16 | 4.1% | -0.60 [-4.08, 2.88] |
| Lehmann 2013 | 45.5 | 21.7 | 42 | 30.2 | 20.1 | 46 | 3.8% | 15.30 [6.54, 24.06] |
| Leventis 2009 | 14.2 | 12.25 | 19 | 13.4 | 7.5 | 50 | 4.0% | 0.80 [-5.09, 6.69] |
| Nimitphong 2013 | 16.13 | 8.8 | 20 | 7.84 | 4.84 | 21 | 4.1% | 8.29 [3.91, 12.67] |
| Oliveri 2015 | 17.85 | 10.82 | 11 | 17.51 | 10.82 | 11 | 3.8% | 0.34 [-8.70, 9.38] |
| Romagnoli (IM) 2008 | 65.3 | 30.2 | 8 | 23.01 | 13.73 | 8 | 2.7% | 42.29 [19.30, 65.28] |
| Romagnoli (O) 2008 | 70.04 | 20.79 | 8 | 25.43 | 16.85 | 8 | 3.0% | 44.61 [26.07, 63.15] |
| Shieh 2016 | 75.12 | 49.84 | 19 | 30.25 | 21.93 | 19 | 2.5% | 44.87 [20.39, 69.35] |
| Thacher 2010 | 72.38 | 24.96 | 10 | 72.38 | 42.43 | 11 | 2.2% | 0.00 [-29.46, 29.46] |
| Trang 1998 | 23.3 | 15.7 | 55 | 13.7 | 11.4 | 17 | 4.0% | 9.60 [2.77, 16.43] |
| Tripkovic (BSKT) 2017 | 30.03 | 36.74 | 67 | 15.96 | 38.19 | 66 | 3.5% | 14.07 [1.33, 26.81] |
| Tripkovic (J) 2017 | 31.22 | 35.08 | 70 | 13.8 | 33.9 | 67 | 3.6% | 17.42 [5.87, 28.97] |
| **Subtotal (95% CI)** | | | **531** | | | **523** | **70.0%** | **11.27 [6.79, 15.74]** |
| Heterogeneity: Tau² = 66.71; Chi² = 94.20, df = 19 (P < 0.00001); I² = 80% | | | | | | | | |
| Test for overall effect: Z = 4.94 (P < 0.00001) | | | | | | | | |
| **1.16.2 Baseline 25(OH)D ≥ 50 nmol/L** | | | | | | | | |
| Binkley (D) 2011 | 22.88 | 8.52 | 16 | 15.08 | 7.35 | 16 | 4.0% | 7.80 [2.29, 13.31] |
| Binkley (M) 2011 | 22.54 | 10.55 | 16 | 9.19 | 6.76 | 16 | 4.0% | 13.35 [7.21, 19.49] |
| Cipriani (O) 2013 | 69.64 | 9.48 | 6 | 0.1 | 0.1 | 6 | 3.9% | 69.54 [61.95, 77.13] |
| Hartwell 1987 | 32.57 | 8.41 | 9 | 13.08 | 10.94 | 9 | 3.8% | 19.49 [10.47, 28.51] |
| Heaney 2011 | 61.93 | 7.91 | 17 | 20.64 | 10.48 | 16 | 4.0% | 41.29 [34.93, 47.65] |
| Itkonen 2016 | 9.3 | 9.42 | 11 | 4.8 | 27.19 | 10 | 3.1% | 4.50 [-13.25, 22.25] |
| Logan 2013 | 1.12 | 22.97 | 32 | -17 | 20.27 | 31 | 3.7% | 18.12 [7.43, 28.81] |
| **Subtotal (95% CI)** | | | **107** | | | **104** | **26.6%** | **25.20 [7.67, 42.72]** |
| Heterogeneity: Tau² = 535.54; Chi² = 216.61, df = 6 (P < 0.00001); I² = 97% | | | | | | | | |
| Test for overall effect: Z = 2.82 (P = 0.005) | | | | | | | | |
| **1.16.3 Others** | | | | | | | | |
| Cipriani (IM) 2013 | 27.96 | 16.22 | 6 | 27.71 | 6.99 | 6 | 3.4% | 0.25 [-13.88, 14.38] |
| **Subtotal (95% CI)** | | | **6** | | | **6** | **3.4%** | **0.25 [-13.88, 14.38]** |
| Heterogeneity: Not applicable | | | | | | | | |
| Test for overall effect: Z = 0.03 (P = 0.97) | | | | | | | | |
| **Total (95% CI)** | | | **644** | | | **633** | **100.0%** | **15.69 [9.46, 21.93]** |
| Heterogeneity: Tau² = 242.77; Chi² = 428.68, df = 27 (P < 0.00001); I² = 94% | | | | | | | | |
| Test for overall effect: Z = 4.94 (P < 0.00001) | | | | | | | | |
| Test for subgroup differences: Chi² = 4.75, df = 2 (P = 0.09), I² = 57.9% | | | | | | | | |

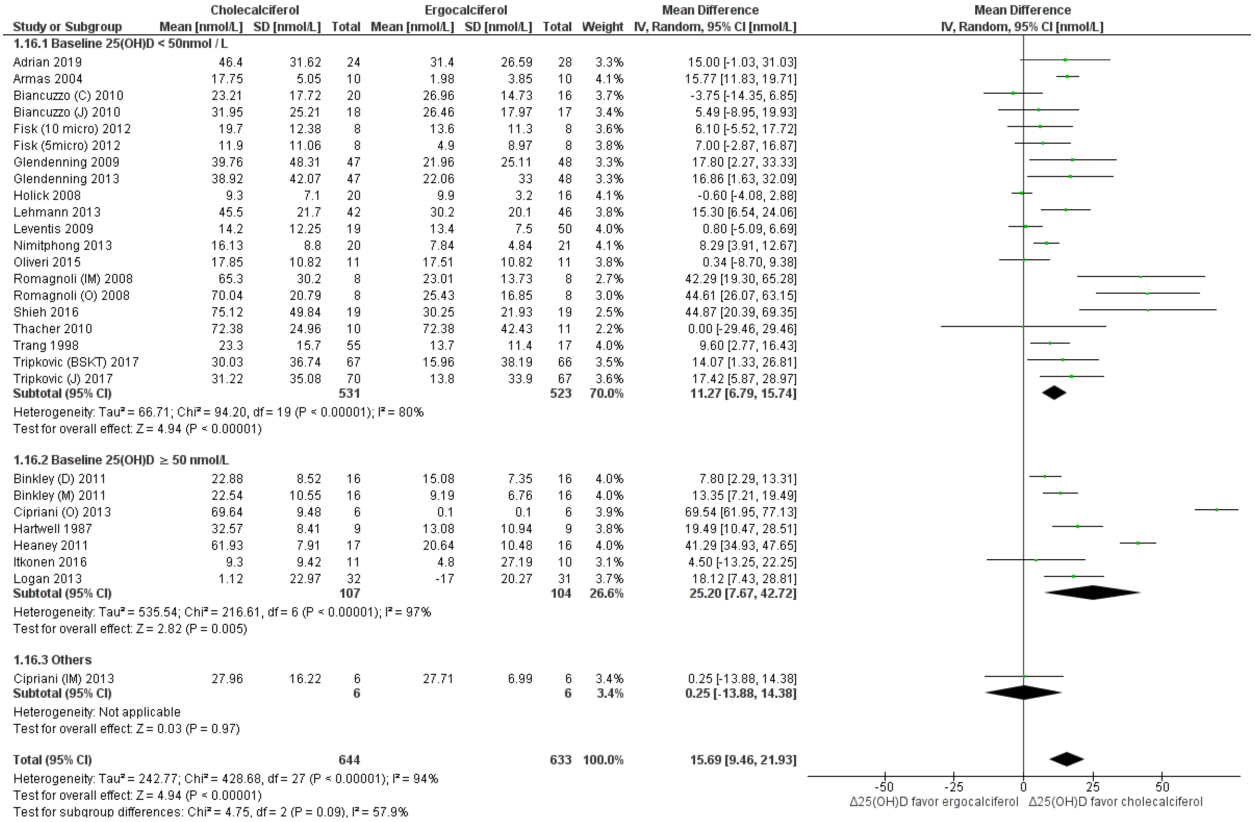

**Supplementary figure S6:** Bubble plot demonstrating the association between "average dose per day" as and mean difference between the 2 interventions

Bubble plot demonstrates the relation between average dose per day and the mean difference in the serum 25(OH)D levels after intervention between the cholecalciferol supplemented group as compared to ergocalciferol supplemented group. The size of the bubble is inversely proportional to the variance of the estimated effect

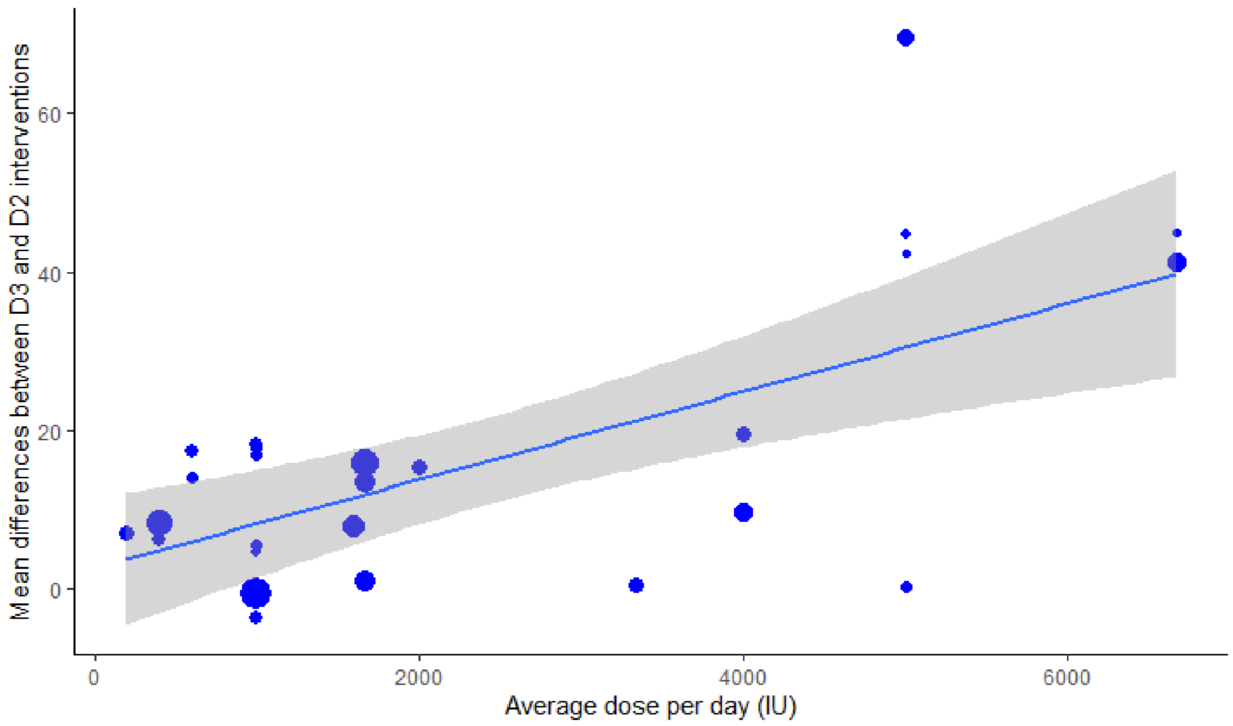

# PRISMA 2009 Checklist

| Section/topic | # | Checklist item | Reported on page # |
|---|---|---|---|
| **TITLE** | | | |
| Title | 1 | Identify the report as a systematic review, meta-analysis, or both. | Yes, 1 |
| **ABSTRACT** | | | |
| Structured summary | 2 | Provide a structured summary including, as applicable: background; objectives; data sources; study eligibility criteria, participants, and interventions; study appraisal and synthesis methods; results; limitations; conclusions and implications of key findings; systematic review registration number. | Yes, 1 |
| **INTRODUCTION** | | | |
| Rationale | 3 | Describe the rationale for the review in the context of what is already known. | Yes, 2 |
| Objectives | 4 | Provide an explicit statement of questions being addressed with reference to participants, interventions, comparisons, outcomes, and study design (PICOS). | Yes, 2 -3 |
| **METHODS** | | | |
| Protocol and registration | 5 | Indicate if a review protocol exists, if and where it can be accessed (e.g., Web address), and, if available, provide registration information including registration number. | Yes, 3 |
| Eligibility criteria | 6 | Specify study characteristics (e.g., PICOS, length of follow-up) and report characteristics (e.g., years considered, language, publication status) used as criteria for eligibility, giving rationale. | Yes, 3 |
| Information sources | 7 | Describe all information sources (e.g., databases with dates of coverage, contact with study authors to identify additional studies) in the search and date last searched. | Yes, 3 |
| Search | 8 | Present full electronic search strategy for at least one database, including any limits used, such that it could be repeated. | Yes, 3 |
| Study selection | 9 | State the process for selecting studies (i.e., screening, eligibility, included in systematic review, and, if applicable, included in the meta-analysis). | Yes, 3 |
| Data collection process | 10 | Describe method of data extraction from reports (e.g., piloted forms, independently, in duplicate) and any processes for obtaining and confirming data from investigators. | Yes, 3 |
| Data items | 11 | List and define all variables for which data were sought (e.g., PICOS, funding sources) and any assumptions and simplifications made. | Yes, 4 |
| Risk of bias in individual studies | 12 | Describe methods used for assessing risk of bias of individual studies (including specification of whether this was done at the study or outcome level), and how this information is to be used in any data synthesis. | Yes, 4 |
| Summary measures | 13 | State the principal summary measures (e.g., risk ratio, difference in means). | Yes, 4 |
| Synthesis of results | 14 | Describe the methods of handling data and combining results of studies, if done, including measures of consistency (e.g., $I^2$) for each meta-analysis. | Yes, 4 |

| Section/topic | # | Checklist item | Reported on page # |
|---|---|---|---|
| Risk of bias across studies | 15 | Specify any assessment of risk of bias that may affect the cumulative evidence (e.g., publication bias, selective reporting within studies). | Yes, 4 |
| Additional analyses | 16 | Describe methods of additional analyses (e.g., sensitivity or subgroup analyses, meta-regression), if done, indicating which were pre-specified. | Yes, 4 - 5 |
| **RESULTS** | | | |
| Study selection | 17 | Give numbers of studies screened, assessed for eligibility, and included in the review, with reasons for exclusions at each stage, ideally with a flow diagram. | Yes, 5 -6 & figure 1 |
| Study characteristics | 18 | For each study, present characteristics for which data were extracted (e.g., study size, PICOS, follow-up period) and provide the citations. | Yes, 5 -6 & table 1 |
| Risk of bias within studies | 19 | Present data on risk of bias of each study and, if available, any outcome level assessment (see item 12). | Yes, 5 -6 & table 2 |
| Results of individual studies | 20 | For all outcomes considered (benefits or harms), present, for each study: (a) simple summary data for each intervention group (b) effect estimates and confidence intervals, ideally with a forest plot. | Yes, 5 - 10 |
| Synthesis of results | 21 | Present results of each meta-analysis done, including confidence intervals and measures of consistency. | Yes, 6 - 15 |
| Risk of bias across studies | 22 | Present results of any assessment of risk of bias across studies (see Item 15). | Yes, 6 -15 |
| Additional analysis | 23 | Give results of additional analyses, if done (e.g., sensitivity or subgroup analyses, meta-regression [see Item 16]). | Yes, 6 -15 |
| **DISCUSSION** | | | |
| Summary of evidence | 24 | Summarize the main findings including the strength of evidence for each main outcome; consider their relevance to key groups (e.g., healthcare providers, users, and policy makers). | Yes, 16 – 17 |
| Limitations | 25 | Discuss limitations at study and outcome level (e.g., risk of bias), and at review-level (e.g., incomplete retrieval of identified research, reporting bias). | Yes, 16 – 17 |
| Conclusions | 26 | Provide a general interpretation of the results in the context of other evidence, and implications for future research. | Yes, 16 – 17 |
| **FUNDING** | | | |
| Funding | 27 | Describe sources of funding for the systematic review and other support (e.g., supply of data); role of funders for the systematic review. | Yes, 18 |

*From:* Moher D, Liberati A, Tetzlaff J, Altman DG, The PRISMA Group (2009). Preferred Reporting Items for Systematic Reviews and Meta-Analyses: The PRISMA Statement. PLoS Med 6(6): e1000097. doi:10.1371/journal.pmed1000097

For more information, visit: www.prisma-statement.org.
